# Associations of Lifestyle, Ambient Air Pollution With Progression of Asthma in Adults: A Comprehensive Analysis of UK Biobank Cohort

**DOI:** 10.3389/ijph.2024.1607640

**Published:** 2024-09-25

**Authors:** Jialu He, Jiahui Wu, Yinan He, Dequan Shen, Xianglong Huang, Xinmeng Yao, Weihong Tang, Guo-Bo Chen, Chengyin Ye

**Affiliations:** ^1^ Department of Epidemiology and Biostatistics, Hangzhou Normal University, Hangzhou, China; ^2^ Department of Health Management, Hangzhou Normal University, Hangzhou, China; ^3^ Department of Pediatrics, Xihu District Hospital of Integrated Traditional Chinese and Western Medicine, Hangzhou, China; ^4^ Department of Gastroenterology, Hangzhou Children’s Hospital, Zhejiang, China; ^5^ Department of General Practice Medicine, Clinical Research Institute, Center of General Practice Medicine, Zhejiang, Provincial Hospital the Affiliated Hospital of Hangzhou Medical College, Hangzhou, China; ^6^ Key Laboratory of Endocrine Gland Diseases of Zhejiang Province, Zhejiang Provincial People’s Hospital, Hangzhou, China

**Keywords:** asthma, socioeconomic status, lifestyle, ambient air pollution, two-pollutant model

## Abstract

**Objectives:**

We aim to investigate the associations between lifestyle, ambient air pollution with crucial outcomes in the progression of adult asthma, including asthma new-onset and asthma hospitalisation.

**Methods:**

176,800 participants were included to assess the prospective association between baseline risk exposures and the subsequent asthma onset, 17,387 participants were used to evaluate asthma hospitalisation. Cox regression models were employed to examine the associations.

**Results:**

In terms of lifestyle factors, the HRs (95% CIs) of the least healthy lifestyle categories for asthma incidence and hospitalization were 1.099 (1.017–1.187) and 1.064 (1.008–1.123), respectively. For pollutants, PM_2.5_, especially the traffic-related PM_2.5_ component, was consistently recognized as a significant risk factor for asthma onset (HR = 1.064, 95% CI: 1.034–1.094) and hospitalisation (HR = 1.031, 95% CI: 1.010–1.052) under various model adjustments. Low socioeconomic status also played a major role in the progression of adult asthma.

**Conclusion:**

Our study provides crucial insights into factors influencing the progression of adult asthma. Monitoring and reducing exposure to air pollution, particularly PM_2.5_, promoting healthier lifestyle, and addressing socioeconomic inequity are important in preventing and managing asthma.

## Introduction

Asthma is a prevalent chronic respiratory disease (CRD) characterized by recurrent wheezing, shortness of breath, chest tightness, cough, and variable airflow limitation [1]. In 2019, asthma affected 262 million people globally and caused over 460,000 deaths [2]. Asthma-related hospital admissions increased by 46.1% from 1999 to 2020 [3]. There is currently no cure for the disease, so identifying risk factors that trigger or accelerate asthma is essential to preventing its onset or exacerbations.

In addition to hereditary component or sensitization to specific allergens, asthma is also affected by various modifiable factors, such as socioeconomic status, lifestyle and environmental exposure [4–7]. Although a systematic review found that 63% of studies link lower socioeconomic position to asthma onset, the evidence is inconclusive [8]. Socioeconomic status (SES), a multidimensional concept reflecting individuals’ or groups’ relative rank and resource allocation, is often treated as a confounding factor in related models [9]. In terms of lifestyle, active or passive smoking, unregular physical activity, unhealthy diet, as well as overall unhealthy lifestyle have been identified as common modifiable risk factors for asthma by some studies [10–13]. Mechanistically, air pollutants likely induce asthma by causing airways oxidative injury, which stimulates inflammation, remodeling, and high sensitization to aeroallergens [14]. But the epidemiological evidence regarding the association between asthma incidence and ambient air pollution (e.g., from traffic, industry) remains inconclusive [15–17]. An European study found that higher exposure to nitrogen oxides (NO_X_), and particulate matter of size 10 μm or less (PM_10_) from traffic was associated with decreased lung function [18]. In urban areas, motor vehicle exhaust is a major source of NO_X_, with approximately 95% emitted as nitric oxide (NO) and a smaller portion as nitrogen dioxide (NO_2_) [19, 20]. NO is highly reactive, and is readily converted to NO_2_ via chemical reactions in the atmosphere, eventually leading to the formation of nitrates. Although NO is unlikely to be a direct causal pollutant of asthma, it has also been recognized as a key precursor of several secondary pollutants and a significant contributor to traffic-related particulate matter of size less 2.5 μm (PM_2.5_) [21]. In addition to the well-studied ambient air pollutants, NO and traffic-related PM_2.5_ deserves great attention in relation to asthma development.

The exacerbation of asthma can be triggered by the factors such as inappropriate use of medicines, viral infection, allergen exposure, and a various modifiable characteristic [22, 23]. Patients with asthma living in more deprived areas likely experienced poorer disease control and higher rates of hospitalisations [24]. Exposure to smoke, which contains potent respiratory irritants, is generally associated with a higher risk of severe asthma exacerbations [25]. Physical inactivity is common among asthma patients, but it is recognized as a significant modifiable risk factor for poor clinical outcomes [26]. A study of 10 European cities found when a causal relationship between traffic-related pollution and asthma is assumed, 15% of all asthma events (episodes of asthma symptoms and asthma hospitalisations) were attributable to air pollution [27]. Some studies indicated a significant positive association between asthma-related emergency room visits and hospital admissions with PM_2.5_, PM_10_, and NO_2_, while others found no consistent associations with PM_10_ and NO_2_ [28–31]. Current findings are controversial and further research is needed to clarify the complex interactions of factors for asthma exacerbation.

There remains a shortage of large-scale population cohort studies to provide solid evidence on the combined association or causal links between lifestyle, ambient air pollutants, and the progression of asthma in adults. To address this gap, we analyze extensive and comprehensive data from the UK Biobank to investigate how lifestyle and air pollutants impact asthma onset and hospitalization. Our study also considers the role of SES and specifically focuses on traffic-related pollutants. With these advantages in data and study design, our large-scale prospective cohort study aims to uncover causal relationships and guide effective preventive strategies and interventions.

## Methods

### Study Design and Population

**FIGURE 1:**
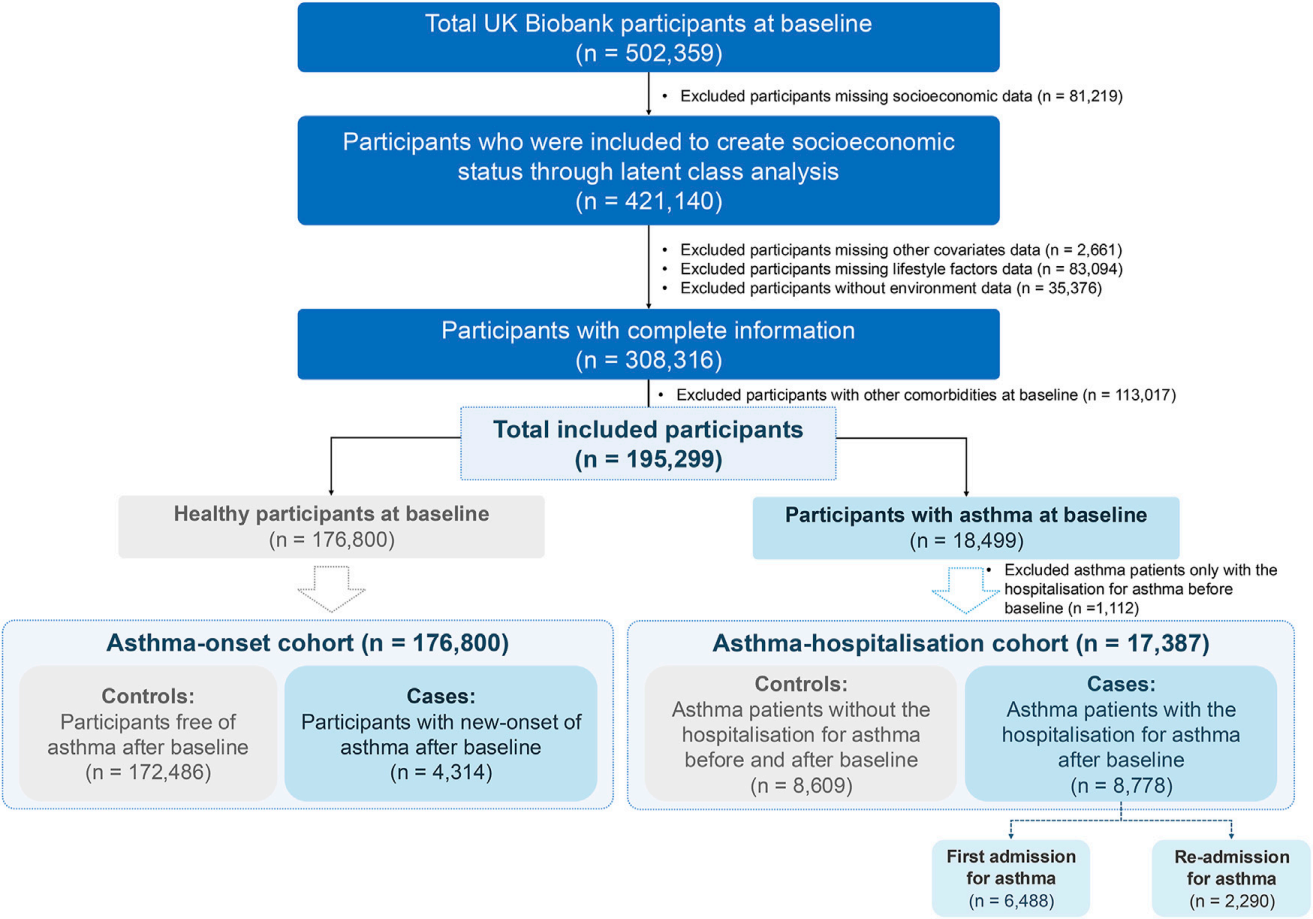
Flowchart of UK Biobank participants. The inclusion and exclusion criteria were applied accordingly to form the two cohorts employed in our study (United Kingdom, 2006–2022).

As a large-scale prospective database, UK Biobank surveyed half a million volunteer participants aged 40–69 years since 2006 and consistently gathered health information and additional exposure data over nearly two decades [32]. The initial assessment visit for participants was conducted between 2006 and 2010, collecting multi-dimensional baseline information of participants including demographic characteristics, socioeconomics, lifestyle, etc., which serving as the source of potential risk factors included in our analysis. We used the participants (421,140) with complete socioeconomic variables data to create the overall SES by latent class analysis (LCA). We applied strict inclusion and exclusion criteria to define our study cohorts (Figure 1). After excluding participants missing covariates, lifestyle, or pollutants data, or with other comorbidities (Supplementary Table S1), and categorizing them based on asthma status at baseline, we created distinct two cohorts for major analyses: 1) the asthma-onset cohort (176,800 healthy individuals) and 2) the asthma-hospitalization cohort (17,387 participants with asthma at baseline, excluding those hospitalized prior).

### Assessment of Outcomes and Follow-Up

This study focused on two primary outcomes during the follow-up: new-onset asthma and asthma hospitalisation. New-onset asthma was defined by the first occurrences category (Category ID: 1712) of UK Biobank, generated by integrating self-report, primary care, hospital inpatient data, and death data. The data of asthma-related hospitalisations (Category ID: 2000) were obtained through linkage to external data providers. Asthma was mapped to a 3-digit code of International Classification of Disease, 10th Revision (ICD-10) code J45 and J46. In our study, follow-up started at each participant’s baseline recruitment (earliest in March 2006, latest in October 2010) and continued until one of the following occurred: an asthma-related outcome (such as the first diagnosis of asthma or asthma hospitalization), death, loss to follow-up, or the end of the follow-up period, which varied by regions (31 October 2022, for England; 31 May 2021, for Wales; and 31 August 2022, for Scotland). Participants who did not experience an asthma-related outcome by the end of the follow-up were labeled as right-censored controls. Follow-up time was measured in months for the survival analysis.

### Assessment of Ambient Air Pollutant Exposures

We used the representative annual concentration estimates of the five air pollutants from the year of 2010 as initial indicators, including fine particulate matter with diameter <2.5 μm (PM_2.5_), particulate matter with diameter between 2.5 μm and 10 μm (PM_coarse_), particulate matter with diameter <10 μm (PM_10_), nitrogen dioxide (NO_2_), nitrogen oxides (NO_X_), which were computed for individual addresses utilizing a Land Use Regression (LUR) model and provided directly by UK Biobank. The LUR model was developed within the framework of the European Study of Cohorts for Air Pollution Effects (ESCAPE) and based on the air pollution measurements and geographic predictor to estimate air pollution exposure for each participant [33]. To avoid bias in our study, we adhered to the principle of not combining or averaging estimates from different air pollution models across years and only used pollutants data from 2010 as a proxy for pollutant levels at the baseline. In addition to the above five pollutants directly provided by UK Biobank, we also calculated and estimated the levels of NO, traffic PM_2.5_, and non-traffic PM_2.5_ to evaluate their impacts on asthma-related outcomes. The term “NO_x_” generally refers to the mixture of NO and NO_2_, as other nitrogen oxides in the atmosphere are not considered to have significant biological effects [34]. So, we calculated NO using the difference between NO_X_ and NO_2_ (i.e., NO = NO_X_ - NO_2_). Then, we constructed both linear and non-linear models to regress PM_2.5_ on NO. As a result, the non-linear spline regression model attained higher R-square values (Supplementary Figure S1). The predicted PM_2.5_ from this model was then defined as “traffic PM_2.5_.” Finally, by subtracting the predicted traffic PM_2.5_ from the total PM_2.5_, we derived the “non-traffic PM_2.5_.”

### Measurements of Demographics, Socioeconomic and Lifestyle Factors

Demographics, socioeconomic and lifestyle variables collected at baseline recruitment were reviewed and carefully aligned with the predetermined asthma-related factors. The final set of baseline variables considered in our study included age, sex, household income, highest level of education, employment status, body mass index (BMI) category, and five lifestyle factors consisting of exposure to second-hand smoke, smoking status, alcohol intake, physical activity and dietary habits (details in Supplementary Table S2).

**FIGURE 2:**
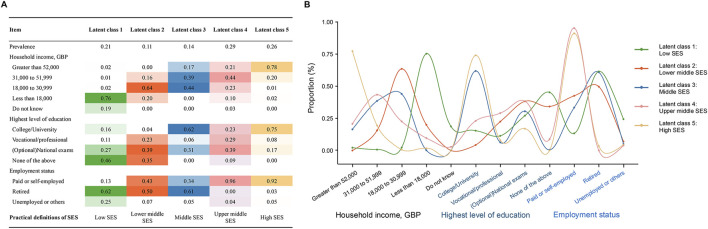
Construct the overall socioeconomic status of the participants through latent class analysis. **(A)** Prevalence of latent classes, and item-related proportion of each latent class; **(B)** Curve plot of each latent class (United Kingdom, 2006–2022).

We used average total household income before tax, highest level of education, and employment status to construct the multidimensional SES for each participant using LCA, as these variables were validated to adequately reflect the individual-level SES [7]. In the UK Biobank, the source and definition of the 3 used variables are listed in Supplementary Table S2. During the LCA to construct the overall individual-level SES variable, the optimal number of latent classes was determined by considering the Bayesian Information Criteria (BIC) (lower values suggest model parsimony), class interpretability (the extent to which additional classes provided clinically relevant information) and class prevalence (i.e., classes with at least 5% of the sample to improve replicability) [35]. Supplementary Figure S2 shows BIC statistics for models with 2–7 latent classes. We finally identified five latent classes: “low SES”, “lower middle SES”, “middle SES”, “upper middle SES” and “high SES”. The characteristics and distribution of the 3 variables within each class are presented in Figure 2.

Five lifestyle factors were used to construct an overall lifestyle score to reflect the overall lifestyle level. For each lifestyle factor, we assigned 0 point for a healthy level and 1 point for an unhealthy level. The lifestyle score was the sum of the points and ranged between 0 and 5. We further divided the participants into three lifestyle categories according to lifestyle score, i.e., most healthy (0–1 point), moderately healthy (2 points), and least healthy (3–5 points) (Supplementary Table S2).

### Statistical Analysis

We summarized baseline characteristics of the two cohorts using descriptive statistics, reporting the mean and standard deviation (SD) for continuous variables and n (%) for categorical variables. The t-test was used to compare means and the chi-square test was used to assess independence between two categorical variables. Spearman correlation coefficients were calculated for each pair of the five air pollutants to assess the degree of correlation between them.

Cox proportional hazard regression models incorporating bidirectional stepwise adjustment were adopted to assess the prospective associations between lifestyle, ambient air pollutants and asthma incidence or asthma hospitalisation. The Schoenfeld residual test was used to test the proportional hazards assumption. The AIC (Akaike Information Criterion) criterion was adopted for stepwise variable selection in the final survival models, ensuring the balance between goodness of fit and model complexity.

In the separate model of lifestyle, we chose to unadjust (Single-1 model) or adjust (Single-full model: was adjusted for age, sex, SES, BMI categories) for other covariates to examine the associations between lifestyle and asthma-related outcomes. In the pollutant models, we set up three single-pollutant models to assess the effect of pollution exposure on the progression of asthma, with hazard ratios calculated per 1 μg/m³ increase in pollutant concentration. Single-1 model was unadjusted for other variables; single-2 model was adjusted for age, sex, SES, and BMI categories; single-full model was further adjusted for lifestyle categories. There were high positive correlations in pairs of pollutants, so two-pollutant models were also constructed (Supplementary Figure S3). Compared with the single-full model, two-pollutant models for PM_2.5_, traffic PM_2.5_, non-traffic PM_2.5_, PM_10_, and PM_coarse_ further adjusted for NO_2_, NO_X_, NO respectively, while two-pollutant models for NO_2_, NO_X_, NO, further adjusted for PM_2.5_, traffic PM_2.5_, non-traffic PM_2.5_, PM_10_, PM_coarse_ respectively. Additionally, the dose-response relationships between pollutants and the risk of asthma incidence or hospitalisation were also assessed by restricted cubic spline regression with three degrees of freedom. The 5th, 50th, and 95th percentiles of the concentration estimate for air pollutants were selected as knots.

Interaction terms of pollutants with other control variates were also introduced in the single-full association models between air pollution and asthma-related outcomes. In subgroup analyses, we also explored the association between primary asthma-related outcomes and some factors (e.g., age, sex, SES and lifestyle categories) in the single-full model, stratified by each quartile of PM_2.5_, to better assess the confounding effect between these factors and PM_2.5_ exposure level.

Additionally, we conducted a series of sensitivity analyses. First, we controlled for the Townsend deprivation index instead of individual SES, the former is a variable reflecting the residence-level socioeconomic status in the UK Biobank, or for both, to evaluate whether the association between residence area level SES or both level SES and asthma-related outcomes remained robust. Second, we focused on participants who had been living at their current address for at least 3 years before baseline, and examined the long-term exposure of air pollution in association with the outcomes of asthma development. Third, we limited the follow-up period to 3 years to examine whether exposure to these potential risk factors triggers an asthma onset and attacks in the short term. Fourth, we excluded events that occurred within the first 3 years of follow-up to reduce potential reverse causation and to explore the relatively long-term effects. R Statistical Software, version 4.3.2 software was used to conduct analyses, and p-*value* < 0.05 was considered statistically significant.

## Results

### Participants’ Characteristics and Distributions

Table 1 summarized characteristics of participants in two cohorts. The asthma-onset cohort (n = 176,800) had 4,314 (2.44%) new-onset asthma cases, and the median time to asthma onset was about 7.33 years (Supplementary Figure S4A). 53.17% individuals aged older than 55 years at baseline and 54.71% were females in the asthma-onset cohort. The asthma-hospitalisation cohort (n = 17,387) had 8,778 cases (50.49%) with asthma hospitalisations, median time to hospitalisation was 5.20 years (Supplementary Figure S4B), 44.87% individuals aged older than 55 years, and 58.14% were females. On both investigated cohorts, more than 60% of participants were categorized into the high/upper-middle SES groups, whereas approximately 10% were classified as low SES. Overall, the lifestyles of the two cohorts are at a relatively healthy level. Nearly 40% of the participants fell into the most healthy lifestyle category, and approximately 25% were classified as having the least healthy lifestyle. The annual average concentration of PM_2.5_, PM_10_, PM_coarse_, NO_2_ and NO_X_ were 9.94 ± 1.04 μg/m^3^, 16.18 ± 1.90 μg/m^3^, 6.41 ± 0.90 μg/m^3^, 26.27 ± 7.55 μg/m^3^ and 43.22 ± 15.18 μg/m^3^ in the asthma-onset cohort, respectively. These average concentrations of pollutants were relatively higher in the asthma-hospitalisation cohort.

**TABLE 1:** List of characteristics and their univariant comparison between events and non-events among two cohorts (United Kingdom, 2006–2022).

Characteristic^a^	Asthma-onset cohort (n = 176,800)				Asthma-hospitalisation cohort (n = 17,387)			
	Total (n = 176,800)	Non-events (n = 172,486)	Events (n = 4,314)	p-value^b^	Total (n = 17,387)	Non-events (n = 8,609)	Events (n = 8,778)	p-value^b^
Demographics								
Age, years, n (%)								
<55	82,790 (46.83)	80,910 (46.91)	1,880 (43.58)	<0.001	9,586 (55.13)	5,316 (61.75)	4,270 (48.64)	<0.001
≥55	94,010 (53.17)	91,576 (53.09)	2,434 (56.42)		7,801 (44.87)	3,293 (38.25)	4,508 (51.36)	
Sex, n (%)								
Male	80,069 (45.29)	78,400 (45.45)	1,669 (38.69)	<0.001	7,278 (41.86)	3,973 (46.15)	3,305 (37.65)	<0.001
Female	96,731 (54.71)	94,086 (54.55)	2,645 (61.31)		10,109 (58.14)	4,636 (53.85)	5,473 (62.35)	
Household income, GBP, n (%)								
Greater than 52,000	54,067 (30.58)	53,040 (30.75)	1,027 (23.81)	<0.001	5,704 (32.81)	3,310 (38.45)	2,394 (27.27)	<0.001
31,000 to 51,999	48,469 (27.41)	47,394 (27.48)	1,075 (24.92)		4,861 (27.96)	2,522 (29.29)	2,339 (26.65)	
18,000 to 30,999	40,572 (22.95)	39,476 (22.89)	1,096 (25.41)		3,606 (20.74)	1,573 (18.27)	2,033 (23.16)	
Less than 18,000	28,305 (16.01)	27,365 (15.87)	940 (21.79)		2,710 (15.59)	1,010 (11.73)	1,700 (19.37)	
Do not know	5,387 (3.05)	5,211 (3.01)	176 (4.07)		506 (2.90)	194 (2.26)	312 (3.55)	
Highest level of education, n (%)								
College/University	70,972 (40.14)	69,487 (40.29)	1,485 (34.43)	<0.001	7,590 (43.65)	4,187 (48.64)	3,403 (38.77)	<0.001
Vocational/professional	29,109 (16.46)	28,325 (16.42)	784 (18.17)		2,900 (16.68)	1,400 (16.26)	1,500 (17.09)	
(Optional)National exams	56,193 (31.78)	54,816 (31.78)	1,377 (31.92)		5,197 (29.89)	2,425 (28.17)	2,772 (31.58)	
None of the above	20,526 (11.62)	19,858 (11.51)	668 (15.48)		1,700 (9.78)	597 (6.93)	1,103 (12.56)	
Employment status, n (%)								
Paid or self-employed	118,801 (67.20)	116,118 (67.32)	2,683 (62.19)	<0.001	12,353 (71.05)	6,622 (76.92)	5,731 (65.29)	<0.001
Retired	46,307 (26.19)	45,067 (26.13)	1,240 (28.74)		3,694 (21.25)	1,399 (16.25)	2,295 (26.14)	
Unemployed or others	11,692 (6.61)	11,301 (6.55)	391 (9.07)		1,340 (7.70)	588 (6.83)	752 (8.57)	
Socioeconomic status, n (%)								
High	55,992 (31.67)	54,921 (31.84)	1,071 (24.83)	<0.001	6,218 (35.76)	3,625 (42.11)	2,593 (29.54)	<0.001
Upper middle	54,854 (31.03)	53,469 (31.00)	1,385 (32.10)		5,450 (31.35)	2,701 (31.37)	2,749 (31.32)	
Middle	24,662 (13.95)	24,113 (13.98)	549 (12.73)		2,221 (12.77)	1,040 (12.08)	1,181 (13.46)	
Lower middle	16,661 (9.42)	16,199 (9.39)	462 (10.71)		1,272 (7.32)	500 (5.81)	772 (8.79)	
Low	24,631 (13.93)	23,784 (13.79)	847 (19.63)		2,226 (12.80)	743 (8.63)	1,483 (16.89)	
BMI, (kg/m^2^), n (%)								
Healthy weight (<25)	63,161 (35.72)	62,053 (35.98)	1,108 (25.68)	<0.001	5,291 (30.43)	2,914 (33.85)	2,377 (27.08)	<0.001
Overweight [25,30)	76,747 (43.41)	74,925 (43.43)	1,822 (42.24)		7,440 (42.79)	3,722 (43.23)	3,718 (42.36)	
Obese (≥30)	36,892 (20.87)	35,508 (20.59)	1,384 (32.08)		4,656 (26.78)	1,973 (22.92)	2,683 (30.56)	
Lifestyle								
Lifestyle factors								
Exposure to second-hand smoke, n (%)								
No	139,116 (78.69)	135,911 (78.80)	3,205 (74.29)	<0.001	13,230 (76.09)	6,682 (77.62)	6,548 (74.60)	<0.001
Yes(≥1 h/w)	37,684 (21.31)	36,575 (21.20)	1,109 (25.71)		4,157 (23.91)	1,927 (22.38)	2,230 (25.40)	
Smoking status, n (%)								
Never	110,443 (62.47)	107,873 (62.54)	2,570 (59.57)	<0.001	10,920 (62.80)	5,488 (63.75)	5,432 (61.88)	0.006
Previous	61,054 (34.53)	59,428 (34.45)	1,626 (37.69)		6,005 (34.54)	2,878 (33.43)	3,127 (35.62)	
Current	5,303 (3.00)	5,185 (3.01)	118 (2.74)		462 (2.66)	243 (2.82)	219 (2.50)	
Alcohol intake, g/d, n (%)								
<1	27,862 (15.76)	26,983 (15.65)	879 (20.38)	<0.001	3,103 (17.85)	1,312 (15.24)	1,791 (20.40)	<0.001
1–8	47,043 (26.61)	45,854 (26.58)	1,189 (27.56)		4,629 (26.62)	2,270 (26.37)	2,359 (26.87)	
8–16	42,400 (23.98)	41,484 (24.05)	916 (21.23)		4,069 (23.40)	2,094 (24.32)	1,975 (22.50)	
≥16	59,495 (33.65)	58,165 (33.72)	1,330 (30.83)		5,586 (32.13)	2,933 (34.07)	2,653 (30.23)	
Physical activity, n (%)								
Regular	99,454 (56.25)	97,110 (56.30)	2,344 (54.33)	0.011	9,523 (54.77)	4,794 (55.69)	4,729 (53.87)	0.017
Unregular	77,346 (43.75)	75,376 (43.70)	1,970 (45.67)		7,864 (45.23)	3,815 (44.31)	4,049 (46.13)	
Dietary habits, n (%)								
Healthy	110,472 (62.48)	107,703 (62.44)	2,769 (64.19)	0.020	10,846 (62.38)	5,342 (62.05)	5,504 (62.70)	0.384
Unhealthy	66,328 (37.52)	64,783 (37.56)	1,545 (35.81)		6,541 (37.62)	3,267 (37.95)	3,274 (37.30)	
Lifestyle score, points, n (%)								
0	24,922 (14.10)	24,362 (14.12)	560 (12.98)	0.104	2,368 (13.62)	1,218 (14.15)	1,150 (13.10)	0.226
1	53,904 (30.49)	52,597 (30.49)	1,307 (30.30)		5,190 (29.85)	2,533 (29.42)	2,657 (30.27)	
2	54,474 (30.81)	53,158 (30.82)	1,316 (30.51)		5,429 (31.22)	2,696 (31.32)	2,733 (31.13)	
3	31,159 (17.62)	30,351 (17.61)	808 (18.72)		3,189 (18.34)	1,578 (18.33)	1,611 (18.35)	
4	10,824 (6.12)	10,544 (6.11)	280 (6.49)		1,055 (6.07)	516 (5.99)	539 (6.15)	
5	1,517 (0.86)	1,474 (0.85)	43 (1.00)		156 (0.90)	68 (0.79)	88 (1.00)	
Lifestyle categories, n (%)								
Most healthy	78,826 (44.58)	76,959 (44.62)	1,867 (43.28)	0.039	7,558 (43.47)	3,751 (43.57)	3,807 (43.37)	0.845
Moderately healthy	54,474 (30.82)	53,158 (30.82)	1,316 (30.50)		5,429 (31.22)	2,696 (31.32)	2,733 (31.13)	
Least healthy	43,500 (24.60)	42,369 (24.56)	1,131 (26.22)		4,400 (25.31)	2,162 (25.11)	2,238 (25.50)	
Ambient air pollutant								
PM_2.5_, (μg/m^3^), mean ± SD	9.94 ± 1.04	9.93 ± 1.04	10.03 ± 1.06	<0.001	9.95 ± 1.04	9.93 ± 1.03	9.98 ± 1.04	0.001
PM_10_, (μg/m^3^), mean ± SD	16.18 ± 1.90	16.18 ± 1.90	16.23 ± 1.84	0.073	16.22 ± 1.90	16.21 ± 1.91	16.23 ± 1.89	0.544
PM_coarse_, (μg/m^3^), mean ± SD	6.41 ± 0.90	6.41 ± 0.90	6.42 ± 0.90	0.853	6.42 ± 0.91	6.42 ± 0.91	6.42 ± 0.91	0.876
NO_2_, (μg/m^3^), mean ± SD	26.27 ± 7.55	26.26 ± 7.55	26.73 ± 7.58	<0.001	26.38 ± 7.47	26.30 ± 7.54	26.46 ± 7.40	0.162
NO_X_, (μg/m^3^), mean ± SD	43.22 ± 15.18	43.20 ± 15.17	44.33 ± 15.53	<0.001	43.41 ± 14.99	43.15 ± 14.96	43.66 ± 15.02	0.026

Note:

Abbreviations: SES, socioeconomic status; BMI, body mass index (calculated as weight in kilograms divided by height in meters squared); PM_2.5_, fine particulate matter with diameter <2.5 μm; PM_10_, particulate matter with diameter <10 μm; PM_coarse_, particulate matter with diameter between 2.5 μm and 10 μm; NO_2_, nitrogen dioxide; NO_X_, nitrogen oxides.

^a^ Mean values ±standard deviation for continuous variables and n (%) for categorical variables.

^b^ The t-test was used to compare the means of two groups. The chi-square test was used to assess the independence between two categorical variables. p-value <0.05 was considered statistically significant.

### Lifestyle, Ambient Air Pollutants and the Incidence of Asthma

**FIGURE 3:**
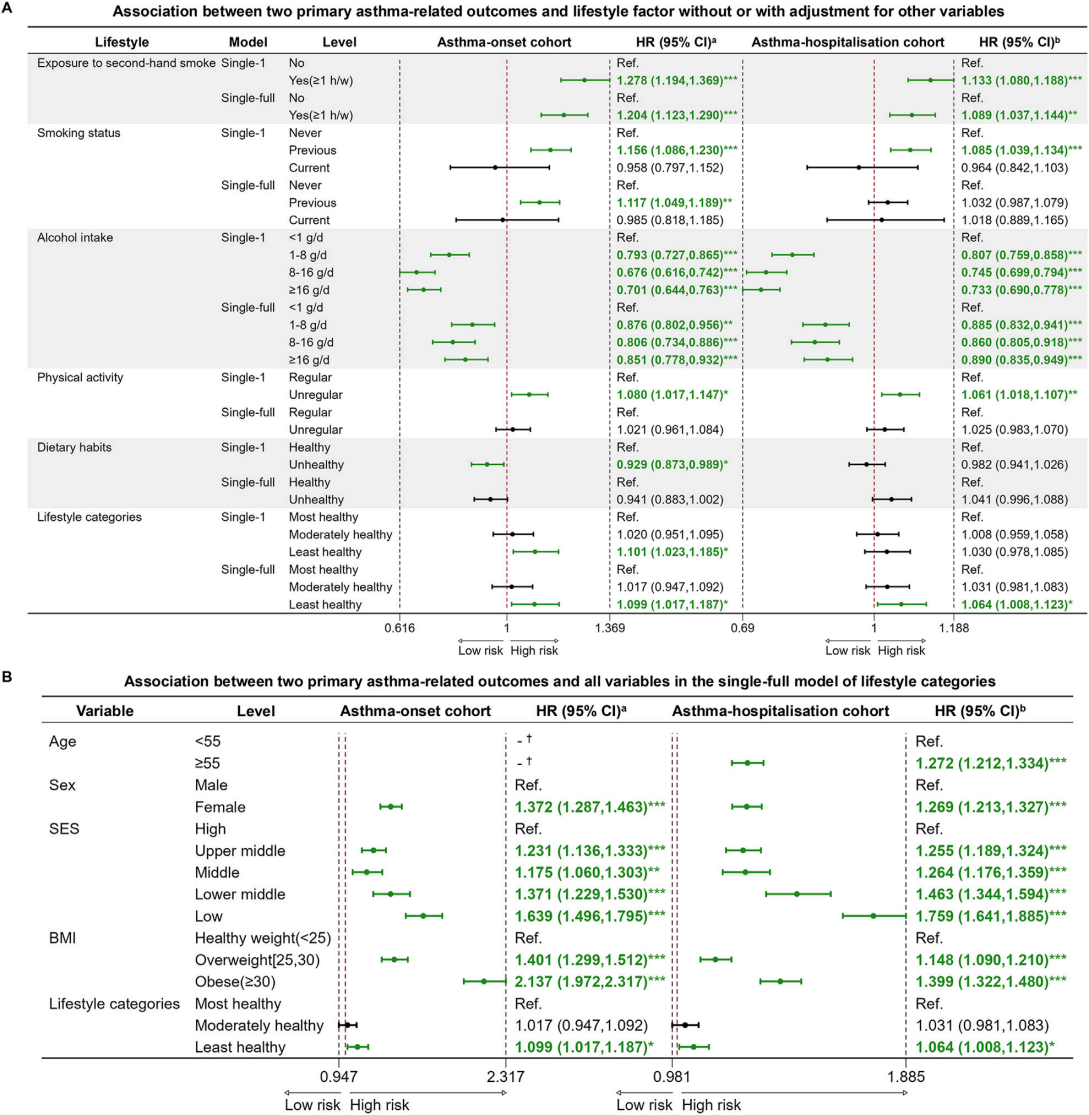
Cox proportional hazard regression models for two primary asthma-related outcomes and lifestyle variables. **(A)** Association between two primary asthma-related outcomes and lifestyle variables without or with adjustment for other variables; **(B)** Association between two primary asthma-related outcomes and all variables in the single-full model of lifestyle categories (United Kingdom, 2006–2022). Note: Abbreviations: HR, hazard ratio; CI, confidence interval; Ref., reference; SES, socioeconomic status; BMI, body mass index (calculated as weight in kilograms divided by height in meters squared). ^a^Hazard ratio (HR) and 95% confidence interval (95% CI) of factors for asthma incidence at follow-up. ^b^Hazard ratio (HR) and 95% confidence interval (95% CI) of factors for asthma hospitalization at follow-up. ^*^p-value <0.05; ^**^ p-value <0.01; ^***^ p-value <0.001. ^†^ The variable didn’t survive in the final stepwise regression model, which was based on the AIC (Akaike Information Criterion) criterion. Single-1 model: unadjusted for other variables. Single-full model: adjusted for age, sex, SES, and BMI categories.

In the univariate analysis, there were significant differences in most variables, except for lifestyle score, PM_coarse_ and PM_10_ (Table 1). In the single-full model of each lifestyle factor for asthma-onset cohort, exposure to second-hand smoke, previous smoking, unregular physical activity were significant risk factors. Exposure to second-hand smoke leaded 1.204 times higher risk of asthma onset compared to no exposure. When considering all lifestyle factors together as a unique lifestyle indicator, the least unhealthy lifestyle would cause 1.099 times higher risk of asthma onset than the healthiest level (Figure 3A). Among the covariates in the fully adjusted model of lifestyle categories, relatively low SES consistently emerged as a significant risk factor for asthma onset, attaining HRs of 1.639 (1.496, 1.795) and 1.371 (1.229, 1.530) than the high SES class, respectively (Figure 3B).

**FIGURE 4:**
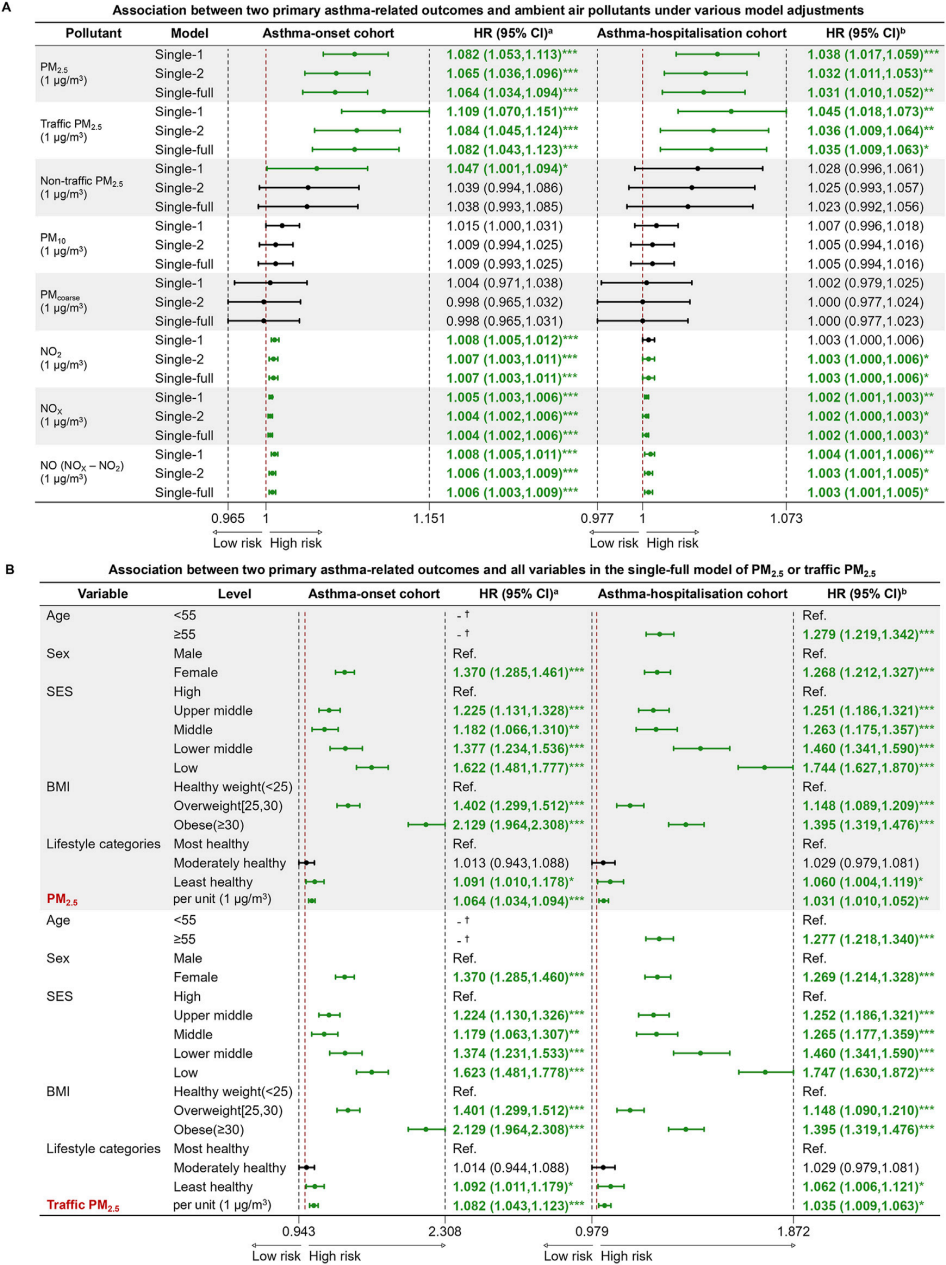
Cox proportional hazard regression models for two primary asthma-related outcomes and ambient air pollutants. **(A)** Association between two primary asthma-related outcomes and ambient air pollutants under various model adjustments; **(B)** Association between two primary asthma-related outcomes and all variables in the single-full model of PM_2.5_ or traffic PM_2.5_ (United Kingdom, 2006–2022). Note: Abbreviations: PM_2.5_, fine particulate matter with diameter <2.5 μm; PM_10_, particulate matter with diameter <10 μm; PM_coarse_, particulate matter with diameter between 2.5 μm and 10 μm; NO_2_, nitrogen dioxide; NO_X_, nitrogen oxides; NO, nitric oxide, NO was determined as the difference between NO_X_ and NO_2_; HR, hazard ratio; CI, confidence interval; Ref., reference; SES, socioeconomic status; BMI, body mass index (calculated as weight in kilograms divided by height in meters squared). ^a^Hazard ratio (HR) and 95% confidence interval (95% CI) of factors for asthma incidence at follow-up. ^b^Hazard ratio (HR) and 95% confidence interval (95% CI) of factors for asthma hospitalization at follow-up. ^*^p-value <0.05; ^**^ p-value <0.01; ^***^ p-value <0.001. ^†^ The variable didn’t survive in the final stepwise regression model, which was based on the AIC (Akaike Information Criterion) criterion. Single-1 model: unadjusted for other variables. Single-2 model: adjusted for age, sex, SES, and BMI categories. Single-full model: adjusted for age, sex, SES, BMI categories and lifestyle categories.

In terms of pollutants, the fully adjusted models showed that PM_2.5_, NO_2_, NO_X_, and NO were significant risk factors for asthma onset, with PM_2.5_ attaining the highest HR of 1.064 (95% CI: 1.034–1.094) per 1 μg/m³ increase in concentration. (Figure 4A). More importantly, traffic PM_2.5_ demonstrated an increased positive effect on asthma incidence (HR = 1.082, 95% CI: 1.043–1.123), compared to the total PM_2.5_. Results from the single-full models for PM_2.5_, traffic PM_2.5_ and non-traffic PM_2.5_ consistently confirmed that female sex, lower SES, higher BMI and an unhealthy lifestyle were significant risk factors of asthma onset (Figure 4B; Supplementary Figure S5). Dose-response relationships between the PM_2.5_ concentrations and asthma incidence exhibited monotonically increasing patterns, with hazard ratios calculated per 1 μg/m³ increment (Supplementary Figure S6). Furthermore, in the fully adjusted two-pollutant models, the association between PM_2.5_ or traffic PM_2.5_ (per 1 μg/m³ increase) and asthma incidence remained robust when adjusted for either NO_2_ or NO (Supplementary Figure S7).

### Lifestyle, Ambient Air Pollutants and the Risk of Asthma Hospitalisation

In the single-full models evaluating the effect of each lifestyle factor for asthma hospitalization, exposure to second-hand smoke and unregular physical activity remained significant risk factors, attaining HRs of 1.089 (1.037, 1.144) and 1.061 (1.018, 1.107), respectively. When using the unique lifestyle indicator, the least healthy lifestyle level caused 1.064 times higher risk of asthma hospitalization compared to the healthiest level (Figure 3A). Consistent with the asthma onset analysis, lower SES was a significant risk factor in the single-full model of lifestyle categories for asthma hospitalization (Figure 3B).

In the fully adjusted models of pollutants, all pollutants showed similar significance to that observed in the asthma-onset cohort. In particular, traffic PM_2.5_ consistently showed a stronger significant association with asthma hospitalization, posing a relatively higher risk per 1 μg/m³ increase in concentration (Figure 4A). In the single-full models for PM_2.5_ and traffic PM_2.5_, the low SES group exhibited HRs of 1.744 (1.627–1.870) and 1.747 (1.630–1.872) for asthma hospitalizations, respectively (Figure 4B), while the least healthy lifestyle group exhibited HRs of 1.060 (1.004–1.119) and 1.062 (1.006–1.121) for hospitalizations compared to the most healthy group. In addition, older individuals, female, participants with unhealthy weight faced higher risk of admission for asthma. After further stratifying the population by first or re-admission for asthma after baseline, both univariate (Supplementary Table S3) and multivariate analyses of lifestyle (Supplementary Figure S8) or pollutants (Supplementary Figures S9, S10) showed that exposure to second-hand smoke, previous smoking, and PM_2.5_ exposure were consistently associated with both first and re-admission for asthma. For asthma hospitalization, the significant effects of PM_2.5_ increased in the two-pollutant models when adjusted for NO_2_, compared to the single-full model (Supplementary Figure S7).

### Subgroup Analyses and Sensitivity Analyses

In the fully adjusted models, male sex interacted with both traffic-related PM_2.5_ and NO, increasing the risk of asthma onset, while female sex significantly interacted with NO and NOx to increase the hospitalization risk (Supplementary Table S4). When stratifying the two cohorts by PM_2.5_ quartiles, HRs of SES and most lifestyle variables were found to be similarly distributed across PM_2.5_ quartile subgroups (Supplementary Table S5, S6; Supplementary Figure S11). However, exposure to second-hand smoke significantly interacted with high PM_2.5_ concentrations (Q4 group), further increasing asthma onset risk (Supplementary Table S5).

When evaluating the individual and combined effects of individual-level SES and the Townsend deprivation index across the fully adjusted models of air pollutants for both cohorts, we observed that the individual-level SES consistently reached higher HRs (Supplementary Table S7). This finding indicates that the LCA-derived individual-level SES is a stronger predictor for both the incidence of asthma and hospitalization rates, as compared to the residence-level SES measure. Supplementary Table S8 illustrated the results of other sensitivity analyses. Most findings remained the same when excluding individuals who resided at the baseline address for less than 3 years before the baseline recruitment and when excluding events that occurred ≤3 years of baseline recruitment. When we limited the follow-up time within 3 years, most risk factors, except for PM_2.5_ and traffic PM_2.5_, became insignificant, implying their cumulative long-term effects on asthma onset and hospitalization.

## Discussion

Based on data from a large UK population-based cohort, this study provided comprehensive, robust and longitudinal evidence on the prospective association between various factors and the progression of asthma in adults. Exposure to secondhand smoke, previous smoking, unregular physical activity and the total lifestyle category were identified as risk factors that could be modifiable. And we found that PM_2.5_ was consistently recognized as a significant risk factor for asthma onset and hospitalisation at follow-up, and notably traffic-related PM_2.5_ emerged as a particularly important category within the total PM_2.5_. The findings of this study provide significant clues and guidance for developing effective prevention and control strategies for asthma.

We observed that aging and being female were associated with a higher risk of asthma development, consistent with findings from previous studies [36, 37]. This could be explained by the vulnerability of immune system and the progressive deterioration of airway function related with aging, as well as hormonal fluctuations in middle-aged women. When SES was treated as a confounding factor in fully adjusted models, it consistently remained a significant risk factor, with HRs exceeding 1.6 for individuals with low SES. Moreover, individuals with low SES and the highest levels of PM_2.5_ exposure have more than twice the risk of developing asthma compared to those with high SES and the lowest levels of PM_2.5_ exposure, which may be related to the health inequalities caused by SES (e.g., greater economic pressures, poorer housing, and limited healthcare resources). The precise role of socioeconomic status in asthma progression remains unclear. The higher asthma prevalence in Western countries may be partly explained by the hygiene hypothesis [8]. But asthma patients from more deprived areas tend to have worse disease control and higher exacerbation rates, likely due to lower use of prophylactic medications [9]. Our study identified overweight and obesity as significant contributors to asthma progression. BMI is an independent risk factor for asthma and the mechanisms that potentially link excess weight to asthma have been discussed elsewhere [38, 39]. A research found that up to 90% of patients experienced reductions in exacerbations, medication use, hospitalizations, and severity scores after losing weight [40]. In this study, BMI was included as a covariate and adjusted for when examining the effects of lifestyle and pollutants on asthma to minimize potential confounding effects related to body weight.

Tobacco smoke, a mixture that includes harmful gases and respirable particulate matter, can affect the onset of asthma by altering airway microbiota [41]. Despite having asthma, patients still commonly experienced second-hand smoke exposure, even those requiring admission [25]. Exposure to second-hand smoke was also identified as a significant risk factor in our study, which was also found to further interact with PM_2.5_ exposure in increasing the risk of new-onset asthma or asthma hospitalisation. Due to the small sample size of current-smoking participants involved in our study, no difference in asthma progression was observed between current smokers and never smokers. Our findings provided evidence that incorporating exercise as a complementary nonpharmacological strategy could be crucial for improving clinical outcomes. Engaging in regular moderate exercise can help lower BMI, reduce airway responsiveness, and enhance pulmonary function. However, physical inactivity remains prevalent among asthma patients, many of whom mistakenly believe that exercise should be avoided [26]. Our study observed a potential protective effect of moderate alcohol intake against asthma onset and hospitalization (not include first hospitalization) [42]. Previous research has shown a U-shaped relationship between alcohol intake and adult asthma incidence, suggesting that moderate alcohol consumption could help regulate asthma development [43]. We also examined the impact of overall lifestyle on asthma progression, least healthy lifestyle was a significant risk factor. We found that individuals with the least healthy lifestyle and highest PM_2.5_ exposure had a 1.385-fold higher risk of asthma onset compared to those with the most healthy lifestyle and lowest exposure, and a 1.176-fold higher risk of asthma hospitalization. This underscores the need to consider both modifiable lifestyle and environmental factors when assessing asthma risk.

In the single-full models of pollutants, we revealed that the risk increased by about 6% for asthma onset and about 3% for asthma hospitalization for each 1 μg/m³ rise in PM_2.5_, with traffic-related PM_2.5_ posing a higher risk than total PM_2.5_. NO_2_, NO_X_, and NO were also significantly associated with asthma progression, though their effects were smaller. These findings were consistent among individuals who lived at the same address for over 3 years or when excluding events within 3 years of baseline recruitment, suggesting a long-term impact of air pollutants and reducing reverse causality concerns. To address pollutant correlations and avoid overestimating individual effects, we used two-pollutant models [44]. Even after adjusting for NO_2_ or NO, PM_2.5_ and traffic PM_2.5_ remained strong contributors to asthma incidence, while nitrogen oxides became primary contributors in models with other PM types. When adjusting for nitrogen oxides, only PM_2.5_ remained a significant factor in asthma hospitalizations. Within sub-cohorts, PM_2.5_ was found to exert more pronounced effects on asthma re-admission compared to first admissions, which was similar to results of other studies [45, 46]. People previously hospitalised for asthma may have compromised airway and immune function, potentially leading to increased sensitivity to pollutants and a higher risk of asthma re-admission. PM_2.5_, with its ability to deposit throughout the respiratory tract, reach the bronchiolar and alveolar regions, and have complex interactions with other pollutants, contribute to oxidative stress and allergic inflammation more easily [47]. In contrast, nitrogen oxides, including nitric oxide and nitrogen dioxide, showed mild airway inflammatory effects only at high levels of exposure. Nitrogen oxides and PM can originate from the same combustion sources, especially the traffic exhaust in residential area, and the former can transform into nitrates and contribute to PM formation [19]. Some studies indicate that the respiratory effects of PM-NO_X_ mixtures may primarily be driven by PM [48, 49]. Therefore, there is considerable debate regarding whether nitrogen oxides directly trigger asthma onset or merely serves as an indicator of the gas-particle mixture originating from traffic sources that contribute to PM formation. In this study, we assessed NO as an indirect means to differentiate between traffic-related and non-traffic-related sources of PM_2.5_. By doing so, the study provides more comprehensive evidence to better understand the risk role of traffic-related pollutants in the progression of asthma, ultimately offering a stronger scientific foundation for public health policies.

The sensitivity analyses suggested that the effects of pollutants on asthma outcomes may be primarily driven by long-term exposure rather than short-term impacts. However, a limitation of our study is the use of 2010 pollutant data as a proxy for baseline exposure, without accounting for changes in pollution levels over time or lag effects. Future studies should incorporate dynamic exposure assessments to better capture both acute and long-term pollution effects on asthma outcomes. While we were unable to include ozone and other pollutants in our study due to the limitations of the UK Biobank’s data acquisition process, we recognize that ozone is an important oxidant that undergoes rapid and complex reactions with NO and NO_2_ in the atmosphere, potentially contributing to asthma development [50–52]. In future studies, acquiring personal pollution data on ozone and other relevant pollutants will be of great importance in further exploring the association between ambient air pollution exposures and asthma-related outcomes.

To conclude, this study provides crucial insights into the factors influencing the progression of adult asthma, especially lifestyle, ambient air pollution, and gives valuable clues for future research and decision-making. Monitoring real-time air pollutant concentrations, especially traffic-related or total PM_2.5_, implementing stricter emission standards, promoting electric vehicle and zero-emission public transport are crucial strategies for reducing pollution emission and exposure [53]. Tobacco smoke is a significant risk factor for asthma, and comprehensive smoke-free laws appears to be effective in improving outcomes. The profiles of high-risk populations for asthma onset and exacerbations help to develop a mobile-based early warning system in mobile to provide exposure alerts and timely interventions (e.g., tips of wearing a facemask, lifestyle adjustments) [54].
