# Supplementary material for: Associations of Lifestyle, Ambient Air Pollution With Progression of Asthma in Adults: A Comprehensive Analysis of UK Biobank Cohort
Source: Int J Public Health. 2024 Sep 25;69:1607640. doi: 10.3389/ijph.2024.1607640 (PMC11461204; doi:10.3389/ijph.2024.1607640)
Supplement: Supplementary file 1 [file DataSheet1.zip › Supplementary Material/Supplementary Material.docx]

**International Journal of Public Health**

**Associations of lifestyle, ambient air pollution with progression of asthma in adults: A comprehensive analysis of UK Biobank cohort**

**Supplementary Material**

**SUPPLEMENTAL TABLES**

**Table S1.** Definition of severe comorbidities used for exclusion criteria (United Kingdom, 2006-2022).

| **Comorbidities** | **Source** | **ICD-10 code** |
| --- | --- | --- |
| Cancer | Self-report and Cancer register | / |
| Respiratory diseases | First occurrences category (Category ID: 1712) | J00 - J44, J47 - J99 |
| Inflammatory disorders |  | G20, G30, G35, K50 - K51, L40, M05 - M07, M32, M45 |
| Cardiovascular disorders |  | I20 - I25, I42 - I43, I46, I50 - I52, I60 - I67, I70 - I71, I73 - I74, Q20 - Q28 |
| Diabetes |  | E10 - E14, O24 |
| HIV |  | B20 - B24 |
| Liver disorders |  | K70 - K77, B18 - B19 |
| Kidney disorders |  | N00 - N29, Q60 - Q63 |

**Table S2.** Definition of demographics and lifestyle variables (United Kingdom, 2006-2022).

| **Variables** | **Definition** | **Source** | **FieldID** |
| --- | --- | --- | --- |
| **Demographics** | | | |
| Age | Two categories: 1. <55 years, 2. ≥55 years. | Some general characteristics of participants, such as date of birth and gender, that were obtained from local NHS Primary Care Trust registries and were known before arrival at the Assessment Centre. | 34 |
|  |  |  | 52 |
|  |  |  | 53 |
| Sex | Two categories: 1. Female, 2. Male. | Some general characteristics of participants, such as date of birth and gender, that were obtained from local NHS Primary Care Trust registries and were known before arrival at the Assessment Centre. | 31 |
| Household income, GBP | Five categories: 1. Greater than 52,000; 2. 31,000 to 51,999; 3. 18,000 to 30,999; 4. Less than 18,000; 5. Do not know. We exclude participants choosing the “prefer not to answer” as missing values in our analysis. | Touchscreen question “What is the average total income before tax received by your HOUSEHOLD”. Possible answers were:  1. Less than 18,000; 2. 18,000 to 30,999; 3. 31,000 to 51,999; 4. 52,000 to 100,000; 5. Greater than 100,000; -1. Do not know; -3. Prefer not to answer | 738 |
| Highest level of education | Four categories: 1. College or University degree; 2. Vocational/professional qualifications, including NVQ or HND or HNC or equivalent, and other professional qualifications; 3. (Optional) National exams, including A levels/AS levels or equivalent, O levels/GCSEs or equivalent, and CSEs or equivalent; 4. None of the above. | Touchscreen question “Which of the following qualifications do you have? (You can select more than one)”. Possible answers were:  1. College or University degree; 2. A levels/AS levels or equivalent; 3. O levels/GCSEs or equivalent; 4. CSEs or equivalent; 5. NVQ or HND or HNC or equivalent; 6. Other professional qualifications e.g.: nursing, teaching; -7. None of the above; -3. Prefer not to answer. | 6138 |
| Employment status | Three categories: 1. Paid or self-employed; 2, Retired; 3. Unemployed or other. | Touchscreen question “Which of the following describes your current situation? (You can select more than one answer)”. Possible answers were:  1. In paid employment or self-employed; 2. Retired; 3. Looking after home and/or family; 4. Unable to work because of sickness or disability; 5. Unemployed; 6. Doing unpaid or voluntary work; 7. Full or part-time student; -7. None of the above; -3.Prefer not to answer. | 6142 |
| BMI categories, (kg/m^2^) | Three categories: 1. Healthy weight (below 24.9); 2. Overweight (between 25 and 29.9); 3. Obese (30 or over) | Body mass index (BMI) was constructed through dividing weight in kilograms by the square of height in meters. | 21001 |
| **Lifestyle** | | | |
| Exposure to second-hand smoke | Exposure to second-hand smoke was defined as exposure ⩾1 h/week to other people’s tobacco smoke at home or outside.  Two categories: 1. No; 2. Yes (≥1 h/w). | Touchscreen question:  1. “At home, about how many hours per WEEK are you exposed to other people's tobacco smoke?”  2. “Outside of your home, about how many hours per WEEK …?” | 1269 |
|  |  |  | 1279 |
| Smoking status | Three categories: 1. Never; 2. Previous; or 3. Current tobacco smokers. | This field summarizes the current/past smoking status of the participant according to the touchscreen question of smoking. | 20116 |
| Alcohol intake | Participants' daily alcohol consumption was calculated based on alcohol intake frequency and weekly or monthly consumption of each alcoholic drink. We assumed that a pint of beer contained 20 g of alcohol, and a glass of other drinks contained 10 g. The total weekly and monthly alcohol consumption was then summed based on these assumptions. Participants who responded with 'do not know' or 'prefer not to answer' to questions about weekly or monthly consumption were coded as missing, except for 'other alcoholic drinks,' where we assigned 0 g. We prioritized participants' reported weekly consumption, and if this was unknown, we used monthly consumption, if available. To estimate daily total consumption, we divided weekly consumption by 7 (or monthly consumption by 30.4375). Median intakes of alcohol in grams per day were calculated for participants in specific categories of alcohol intake frequency, including “daily or almost daily,” “three or four times a week,” “once or twice a week,” “one to three times per month,” and “Special occasions only.” For participants with unknown grams/day of alcohol but reporting one of these top five frequency categories, we assigned them the median value (g/day) from their category. Participants with unknown grams/day of alcohol but reporting an overall frequency of 'never' were assigned to '0 g/day'.  Four categories: 1. <1 g/d; 2.1-8 g/d; 3. 8-16 g/d; 4. ≥16 g/d. | Touchscreen question:  1. “About how often do you drink alcohol?”  2. “In an average WEEK, how many glasses of RED wine would you drink? “  3. “In an average WEEK, how many glasses of WHITE wine or champagne ...?”  4. “In an average WEEK, how many pints of beer or cider ...?”  5. “In an average WEEK, how many measures of spirits or liqueurs ...?”  6. “In an average WEEK, how many glasses of fortified wine ...?”  7. “In an average WEEK, how many glasses of other alcoholic drinks ...?”  8. “In an average MONTH, how many glasses of RED wine ...?”  9. “In an average MONTH, how many glasses of WHITE wine or champagne ...?”  10. “In an average MONTH, how many pints of beer or cider ...?”  11. “In an average MONTH, how many measures of spirits or liqueurs ...?”  12. “In an average MONTH, how many glasses of fortified wine ...?”  13. “In an average MONTH, how many glasses of other alcoholic drinks ...?” | 1558 |
|  |  |  | 1568 |
|  |  |  | 1578 |
|  |  |  | 1588 |
|  |  |  | 1598 |
|  |  |  | 1608 |
|  |  |  | 5364 |
|  |  |  | 4407 |
|  |  |  | 4418 |
|  |  |  | 4429 |
|  |  |  | 4440 |
|  |  |  | 4451 |
| Physical activity | Regular physical activity was defined as ≥150 minutes moderate activity per week OR ≥ 75 minutes vigorous activity per week OR ≥150 minutes mixed (moderate + vigorous) activity.  Two categories: 1. Regular; 2. Unregular. | Touchscreen question:  1. “In a typical WEEK, on how many days ... 10 minutes or more of moderate physical activities?”  2. “How many minutes ... moderate activities on a typical DAY?”  3. “In a typical WEEK, how many days ... 10 minutes or more of vigorous physical activity?”  4. “How many minutes ... vigorous activities on a typical DAY?” | 884 |
|  |  |  | 894 |
|  |  |  | 904 |
|  |  |  | 914 |
| Dietary habits | Healthy dietary habits was defined as meting ≥ 3 of 5 criteria below: vegetable intake ≥ 4 tablespoons/day; fruit intake ≥ 3 pieces/day; fish intake ≥ 2/week; unprocessed red meat intake ≤ 2/week; and processed meat intake ≤ 2/week  Two categories: 1. Healthy; 2. Unhealthy. | Touchscreen question:   1. “On average how many heaped tablespoons of COOKED vegetables would you eat per DAY?” 2. “On average how many heaped tablespoons of SALAD or RAW vegetables …?” 3. “About how many pieces of FRESH fruit …?” 4. “About how many pieces of DRIED fruit …?” 5. “How often do you eat oily fish?” 6. “How often do you eat other types of fish?” 7. “How often do you eat beef?” 8. “How often do you eat lamb/mutton?” 9. “How often do you eat pork?” 10. “How often do you eat processed meats?” | 1289 |
|  |  |  | 1299 |
|  |  |  | 1309 |
|  |  |  | 1319 |
|  |  |  | 1329 |
|  |  |  | 1339 |
|  |  |  | 1369 |
|  |  |  | 1379 |
|  |  |  | 1389 |
|  |  |  | 1349 |
| Lifestyle score | We used five lifestyle factors to construct an overall lifestyle score, including exposure to second-hand smoke (“No” was defined as healthy), smoking status (“Never” was defined as healthy), alcohol intake (“<16 g/d” was defined as healthy), physical activity (“Regular” was defined as healthy), dietary habits (“Healthy” was defined as healthy). For each lifestyle factor, we assigned 0 point for a healthy level and 1 point for an unhealthy level. The lifestyle score was the sum of the points and ranged between 0 and 5. | | |
| Lifestyle categories | We divided the participants into three groups according to lifestyle score, i.e., most healthy (0-1 point), moderately healthy (2 points), and least healthy (3-5 points).  Three categories: 1. Most healthy; 2. Moderately healthy; 3. Least healthy. | | |

**Table S3.** Univariant comparison of characteristics between non-events and events among the two subsets of asthma-hospitalisation cohort (United Kingdom, 2006-2022).

| **Characteristic^*^** | **First admission for asthma (n = 15,097)** | | | | **Re-admission for asthma (n = 10,899)** | | | |
| --- | --- | --- | --- | --- | --- | --- | --- | --- |
|  | **Total**  **(n = 15,097)** | **Non-events**  **(n = 8,609)** | **Events**  **(n = 6,488)** | **p-value^†^** | **Total**  **(n = 10,899)** | **Non-events**  **(n = 8,609)** | **Events**  **(n = 2,290)** | **p-value^†^** |
| Demographics |  |  |  |  |  |  |  |  |
| Age,years, n(%) |  |  |  |  |  |  |  |  |
| <55 | 8,542 (56.58) | 5,316 (61.75) | 3,226 (49.72) | <0.001 | 6,360 (58.35) | 5,316 (61.75) | 1,044 (45.59) | <0.001 |
| ≥55 | 6,555 (43.42) | 3,293 (38.25) | 3,262 (50.28) |  | 4,539 (41.65) | 3,293 (38.25) | 1,246 (54.41) |  |
| Sex, n(%) |  |  |  |  |  |  |  |  |
| Male | 6,570 (43.52) | 3,973 (46.15) | 2,597 (40.03) | <0.001 | 4,681 (42.95) | 3,973 (46.15) | 708 (30.92) | <0.001 |
| Female | 8,527 (56.48) | 4,636 (53.85) | 3,891 (59.97) |  | 6,218 (57.05) | 4,636 (53.85) | 1,582 (69.08) |  |
| Household income, GBP, n(%) |  |  |  |  |  |  |  |  |
| Greater than 52,000 | 5,217 (34.56) | 3,310 (38.45) | 1,907 (29.39) | <0.001 | 3,797 (34.84) | 3,310 (38.45) | 487 (21.27) | <0.001 |
| 31,000 to 51,999 | 4,288 (28.40) | 2,522 (29.29) | 1,766 (27.22) |  | 3,095 (28.40) | 2,522 (29.29) | 573 (25.02) |  |
| 18,000 to 30,999 | 3,047 (20.18) | 1,573 (18.27) | 1,474 (22.72) |  | 2,132 (19.56) | 1,573 (18.27) | 559 (24.41) |  |
| Less than 18,000 | 2,141 (14.18) | 1,010 (11.73) | 1,131 (17.43) |  | 1,579 (14.49) | 1,010 (11.73) | 569 (24.85) |  |
| Do not know | 404 (2.68) | 194 (2.25) | 210 (3.24) |  | 296 (2.72) | 194 (2.25) | 102 (4.45) |  |
| Highest level of education, n(%) |  |  |  |  |  |  |  |  |
| College/University | 6,841 (45.31) | 4,187 (48.64) | 2,654 (40.91) | <0.001 | 4,936 (45.29) | 4,187 (48.64) | 749 (32.71) | <0.001 |
| Vocational/professional | 2,502 (16.57) | 1,400 (16.26) | 1,102 (16.99) |  | 1,798 (16.50) | 1,400 (16.26) | 398 (17.38) |  |
| (Optional)National exams | 4,441 (29.42) | 2,425 (28.17) | 2,016 (31.07) |  | 3,181 (29.19) | 2,425 (28.17) | 756 (33.01) |  |
| None of the above | 1,313 (8.70) | 597 (6.93) | 716 (11.04) |  | 984 (9.03) | 597 (6.93) | 387 (16.90) |  |
| Employment status, n(%) |  |  |  |  |  |  |  |  |
| Paid or self-employed | 11,010 (72.93) | 6,622 (76.92) | 4,388 (67.63) | <0.001 | 7,965 (73.08) | 6,622 (76.92) | 1,343 (58.65) | <0.001 |
| Retired | 3,028 (20.06) | 1,399 (16.25) | 1,629 (25.11) |  | 2,065 (18.95) | 1,399 (16.25) | 666 (29.08) |  |
| Unemployed or others | 1,059 (7.01) | 588 (6.83) | 471 (7.26) |  | 869 (7.97) | 588 (6.83) | 281 (12.27) |  |
| Socioeconomic status, n(%) |  |  |  |  |  |  |  |  |
| High | 5,665 (37.52) | 3,625 (42.11) | 2,040 (31.44) | <0.001 | 4,178 (38.33) | 3,625 (42.11) | 553 (24.15) | <0.001 |
| Upper middle | 4,764 (31.56) | 2,701 (31.37) | 2,063 (31.80) |  | 3,387 (31.08) | 2,701 (31.37) | 686 (29.96) |  |
| Middle | 1,949 (12.91) | 1,040 (12.08) | 909 (14.01) |  | 1,312 (12.04) | 1,040 (12.08) | 272 (11.88) |  |
| Lower middle | 1,024 (6.78) | 500 (5.81) | 524 (8.08) |  | 748 (6.86) | 500 (5.81) | 248 (10.83) |  |
| Low | 1,695 (11.23) | 743 (8.63) | 952 (14.67) |  | 1,274 (11.69) | 743 (8.63) | 531 (23.19) |  |
| BMI, (kg/m2), n(%) |  |  |  |  |  |  |  |  |
| Healthy weight(<25) | 4,775 (31.63) | 2,914 (33.85) | 1,861 (28.68) | <0.001 | 3,430 (31.47) | 2,914 (33.85) | 516 (22.53) | <0.001 |
| Overweight[25,30) | 6,491 (43.00) | 3,722 (43.23) | 2,769 (42.68) |  | 4,671 (42.86) | 3,722 (43.23) | 949 (41.44) |  |
| Obese(≥30) | 3,831 (25.38) | 1,973 (22.92) | 1,858 (28.64) |  | 2,798 (25.67) | 1,973 (22.92) | 825 (36.03) |  |
| Lifestyle |  |  |  |  |  |  |  |  |
| Lifestyle factors |  |  |  |  |  |  |  |  |
| Exposure to second-hand smoke, n(%) |  |  |  |  |  |  |  |  |
| No | 11,568 (76.62) | 6,682 (77.62) | 4,886 (75.31) | 0.001 | 8,344 (76.56) | 6,682 (77.62) | 1,662 (72.58) | <0.001 |
| Yes(≥1 h/w) | 3,529 (23.38) | 1,927 (22.38) | 1,602 (24.69) |  | 2,555 (23.44) | 1,927 (22.38) | 628 (27.42) |  |
| Smoking status, n(%) |  |  |  |  |  |  |  |  |
| Never | 9,509 (62.99) | 5,488 (63.75) | 4,021 (61.98) | 0.014 | 6,899 (63.30) | 5,488 (63.75) | 1,411 (61.62) | 0.095 |
| Previous | 5,185 (34.34) | 2,878 (33.43) | 2,307 (35.56) |  | 3,698 (33.93) | 2,878 (33.43) | 820 (35.81) |  |
| Current | 403 (2.67) | 243 (2.82) | 160 (2.47) |  | 302 (2.77) | 243 (2.82) | 59 (2.58) |  |
| Alcohol intake, g/d, n(%) |  |  |  |  |  |  |  |  |
| <1 | 2,489 (16.49) | 1,312 (15.24) | 1,177 (18.14) | <0.001 | 1,926 (17.67) | 1,312 (15.24) | 614 (26.81) | <0.001 |
| 1-8 | 4,006 (26.54) | 2,270 (26.37) | 1,736 (26.76) |  | 2,893 (26.54) | 2,270 (26.37) | 623 (27.21) |  |
| 8-16 | 3,612 (23.93) | 2,094 (24.32) | 1,518 (23.40) |  | 2,551 (23.41) | 2,094 (24.32) | 457 (19.96) |  |
| ≥16 | 4,990 (33.05) | 2,933 (34.07) | 2,057 (31.70) |  | 3,529 (32.38) | 2,933 (34.07) | 596 (26.03) |  |
| Physical activity, n(%) |  |  |  |  |  |  |  |  |
| Regular | 8,322 (55.12) | 4,794 (55.69) | 3,528 (54.38) | 0.113 | 5,995 (55.01) | 4,794 (55.69) | 1,201 (52.45) | 0.006 |
| Unregular | 6,775 (44.88) | 3,815 (44.31) | 2,960 (45.62) |  | 4,904 (44.99) | 3,815 (44.31) | 1,089 (47.55) |  |
| Dietary habits, n(%) |  |  |  |  |  |  |  |  |
| Healthy | 9,400 (62.26) | 5,342 (62.05) | 4,058 (62.55) | 0.546 | 6,788 (62.28) | 5,342 (62.05) | 1,446 (63.14) | 0.350 |
| Unhealthy | 5,697 (37.74) | 3,267 (37.95) | 2,430 (37.45) |  | 4,111 (37.72) | 3,267 (37.95) | 844 (36.86) |  |
| Lifestyle score, points, n(%) |  |  |  |  |  |  |  |  |
| 0 | 2,064 (13.67) | 1,218 (14.15) | 846 (13.04) | 0.102 | 1,522 (13.96) | 1,218 (14.15) | 304 (13.28) | 0.479 |
| 1 | 4,507 (29.85) | 2,533 (29.42) | 1,974 (30.43) |  | 3,216 (29.51) | 2,533 (29.42) | 683 (29.83) |  |
| 2 | 4,687 (31.05) | 2,696 (31.32) | 1,991 (30.69) |  | 3,438 (31.54) | 2,696 (31.32) | 742 (32.40) |  |
| 3 | 2,797 (18.53) | 1,578 (18.33) | 1,219 (18.79) |  | 1,970 (18.08) | 1,578 (18.33) | 392 (17.12) |  |
| 4 | 903 (5.98) | 516 (5.99) | 387 (5.96) |  | 668 (6.13) | 516 (5.99) | 152 (6.64) |  |
| 5 | 139 (0.92) | 68 (0.79) | 71 (1.09) |  | 85 (0.78) | 68 (0.79) | 17 (0.74) |  |
| Lifestyle categories, n(%) |  |  |  |  |  |  |  |  |
| Most healthy | 6,571 (43.53) | 3,751 (43.57) | 2,820 (43.46) | 0.531 | 4,738 (43.47) | 3,751 (43.57) | 987 (43.10) | 0.594 |
| Moderately healthy | 4,687 (31.05) | 2,696 (31.32) | 1,991 (30.69) |  | 3,438 (31.54) | 2,696 (31.32) | 742 (32.40) |  |
| Least healthy | 3,839 (25.43) | 2,162 (25.11) | 1,677 (25.85) |  | 2,723 (24.98) | 2,162 (25.11) | 561 (24.50) |  |
| Ambient air pollutant |  |  |  |  |  |  |  |  |
| PM_2.5_, (μg/m^3^), mean ± SD | 9.94 ± 1.03 | 9.93 ± 1.03 | 9.97 ± 1.03 | 0.028 | 9.95 ± 1.04 | 9.93 ± 1.03 | 10.02 ± 1.04 | <0.001 |
| PM_10_, (μg/m^3^), mean ± SD | 16.21 ± 1.90 | 16.21 ± 1.91 | 16.21 ± 1.89 | 0.924 | 16.22 ± 1.90 | 16.21 ± 1.91 | 16.27 ± 1.90 | 0.191 |
| PM_coarse_, (μg/m^3^), mean ± SD | 6.42 ± 0.91 | 6.42 ± 0.91 | 6.42 ± 0.91 | 0.820 | 6.42 ± 0.91 | 6.42 ± 0.91 | 6.43 ± 0.92 | 0.948 |
| NO_2_, (μg/m^3^), mean ± SD | 26.33 ± 7.48 | 26.30 ± 7.54 | 26.35 ± 7.39 | 0.688 | 26.40 ± 7.52 | 26.30 ± 7.54 | 26.77 ± 7.43 | 0.008 |
| NO_X_, (μg/m^3^), mean ± SD | 43.25 ± 14.89 | 43.15 ± 14.96 | 43.38 ± 14.80 | 0.357 | 43.43 ± 15.10 | 43.15 ± 14.96 | 44.45 ± 15.61 | <0.001 |

Note:

Abbreviations: SES, socioeconomic status; BMI, body mass index (calculated as weight in kilograms divided by height in meters squared); PM_2.5_, fine particulate matter with diameter < 2.5 μm; PM_10_, particulate matter with diameter < 10 μm; PM_coarse_, particulate matter with diameter between 2.5 μm and 10 μm; NO_2_, nitrogen dioxide; NO_X_, nitrogen oxides.

^*^Mean values ± standard deviation for continuous variables and n (%) for categorical variables.

**^†^**The t-test was used to compare the means of two groups. The chi-square test was used to assess the independence between two categorical variables. p-value < 0.05 was considered statistically significant.

**Table S4.** The hazard ratio (HR) and 95% confidence interval (95% CI) of major pollutants for two primary asthma-related outcomes in the single-full model, stratified by age, sex, SES, lifestyle categories (United Kingdom, 2006-2022).

| **Subgroup** | **No. of events / Total (%)** | **PM_2.5_, (μg/m^3^)** | **Traffic PM_2.5_, (μg/m^3^)** | **Non-traffic PM_2.5_, (μg/m^3^)** | **NO_2_, (μg/m^3^)** | **NO_X_, (μg/m^3^)** | **NO, (μg/m^3^)** |
| --- | --- | --- | --- | --- | --- | --- | --- |
| **Asthma-onset cohort** |  |  |  |  |  |  |  |
| **Age** |  |  |  |  |  |  |  |
| <55 | 1,880 / 82,790 (2.27%) | 1.056 (1.013,1.101)^*^ | 1.075 (1.018,1.136)^*^ | 1.031 (0.965,1.102) | 1.005 (0.999,1.011) | 1.003 (1.000,1.006)^*^ | 1.005 (1.000,1.010)^*^ |
| ≥55 | 2,434 / 94,010 (2.59%) | 1.068 (1.028,1.110)^**^ | 1.085 (1.033,1.141)^**^ | 1.041 (0.980,1.104) | 1.009 (1.003,1.014)^**^ | 1.004 (1.002,1.007)^**^ | 1.007 (1.002,1.011)^**^ |
| p-value for interaction**^†^** |  | 0.844 | 0.939 | 0.941 | 0.472 | 0.548 | 0.682 |
| **Sex** |  |  |  |  |  |  |  |
| Male | 1,669 / 80,069 (2.08%) | 1.068 (1.021,1.117)^**^ | 1.132 (1.068,1.199)^***^ | 0.983 (0.915,1.055) | 1.008 (1.002,1.014)^*^ | 1.005 (1.002,1.008)^***^ | 1.010 (1.005,1.014)^***^ |
| Female | 2,645 / 96,731 (2.73%) | 1.060 (1.022,1.099)^**^ | 1.049 (1.000,1.100) | 1.074 (1.015,1.136)^*^ | 1.007 (1.001,1.012)^*^ | 1.003 (1.000,1.005)^*^ | 1.003 (0.999,1.007) |
| p-value for interaction**^†^** |  | 0.658 | **0.033** | 0.068 | 0.646 | 0.145 | **0.033** |
| **SES** |  |  |  |  |  |  |  |
| High | 1,071 / 55,992 (1.91 %) | 1.066 (1.009,1.126)^*^ | 1.081 (1.005,1.163)^*^ | 1.052 (0.962,1.150) | 1.005 (0.998,1.013) | 1.003 (0.999,1.006) | 1.005 (0.998,1.011) |
| Upper middle | 1,385 / 54,854 (2.52%) | 1.027 (0.975,1.081) | 1.064 (0.996,1.136) | 0.974 (0.901,1.054) | 1.003 (0.996,1.010) | 1.002 (0.999,1.006) | 1.004 (0.999,1.010) |
| Middle | 549 / 24,662 (2.23%) | 1.123 (1.042,1.210)^**^ | 1.095 (0.990,1.212) | 1.180 (1.045,1.333)^**^ | 1.013 (1.003,1.023)^*^ | 1.006 (1.001,1.010)^*^ | 1.008 (1.000,1.016) |
| Lower middle | 1,385 / 54,854 (2.52%) | 1.060 (0.966,1.163) | 1.059 (0.941,1.192) | 1.049 (0.914,1.205) | 1.005 (0.992,1.019) | 1.003 (0.997,1.010) | 1.006 (0.995,1.017) |
| Low | 847 / 24,631 (3.44%) | 1.077 (1.011,1.147)^*^ | 1.112 (1.024,1.208)^*^ | 1.029 (0.933,1.135) | 1.012 (1.003,1.021)^*^ | 1.006 (1.001,1.010)^**^ | 1.008 (1.001,1.015)^*^ |
| p-value for interaction**^†^** |  | 0.272 | 0.375 | 0.564 | 0.076 | 0.112 | 0.213 |
| **Lifestyle categories** |  |  |  |  |  |  |  |
| Most healthy | 1,867 / 78,826 (2.37 %) | 1.034 (0.989,1.081) | 1.051 (0.992,1.113) | 1.009 (0.942,1.080) | 1.004 (0.998,1.010) | 1.002 (0.999,1.005) | 1.003 (0.998,1.008) |
| Moderately healthy | 1,316 / 54,474 (2.42 %) | 1.097 (1.044,1.154)^***^ | 1.108 (1.037,1.184)^**^ | 1.082 (0.999,1.171) | 1.010 (1.003,1.017)^**^ | 1.005 (1.002,1.008)^**^ | 1.008 (1.002,1.014)^**^ |
| Least healthy | 1,131 / 43,500 (2.60 %) | 1.077 (1.021,1.136)^**^ | 1.106 (1.031,1.186)^**^ | 1.039 (0.954,1.132) | 1.010 (1.003,1.018)^**^ | 1.005 (1.001,1.008)^**^ | 1.008 (1.002,1.013)^*^ |
| p-value for interaction**^†^** |  | 0.173 | 0.203 | 0.495 | 0.154 | 0.175 | 0.233 |
| **Asthma-hospitalisation cohort** |  |  |  |  |  |  |  |
| **Age** |  |  |  |  |  |  |  |
| <55 | 4,270 / 9,586 (44.54%) | 1.030 (1.001,1.060)^*^ | 1.041 (1.003,1.080)^*^ | 1.014 (0.970,1.060) | 1.004 (1.000,1.008) | 1.002 (1.000,1.004)^*^ | 1.003 (1.000,1.006) |
| ≥55 | 4,508 / 7,801 (57.79%) | 1.034 (1.005,1.063)^*^ | 1.033 (0.995,1.072) | 1.035 (0.992,1.081) | 1.003 (0.999,1.007) | 1.002 (1.000,1.004) | 1.003 (1.000,1.006) |
| p-value for interaction**^†^** |  | 0.946 | 0.619 | 0.598 | 0.599 | 0.693 | 0.811 |
| **Sex** |  |  |  |  |  |  |  |
| Male | 3,305 / 7,278 (45.41%) | 1.023 (0.989,1.057) | 1.011 (0.969,1.055) | 1.038 (0.986,1.093) | 1.000 (0.996,1.005) | 1.000 (0.998,1.002) | 1.000 (0.996,1.004) |
| Female | 5,473 / 10,109 (54.14%) | 1.035 (1.010,1.062)^**^ | 1.051 (1.016,1.087)^**^ | 1.015 (0.977,1.056) | 1.005 (1.001,1.008)^*^ | 1.003 (1.001,1.004)^**^ | 1.005 (1.002,1.008)^**^ |
| p-value for interaction**^†^** |  | 0.339 | 0.072 | 0.567 | 0.068 | **0.025** | **0.021** |
| **SES** |  |  |  |  |  |  |  |
| High | 2,593 / 6,218 (41.70 %) | 1.038 (1.002,1.075)^*^ | 1.041 (0.992,1.092) | 1.039 (0.981,1.100) | 1.002 (0.997,1.006) | 1.001 (0.999,1.004) | 1.003 (0.999,1.007) |
| Upper middle | 2,749 / 5,450 (50.44%) | 1.023 (0.985,1.062) | 1.040 (0.992,1.091) | 0.995 (0.940,1.053) | 1.004 (0.998,1.009) | 1.002 (0.999,1.004) | 1.003 (0.999,1.007) |
| Middle | 1,181 / 2,221 (53.17%) | 0.987 (0.933,1.043) | 0.993 (0.921,1.071) | 0.977 (0.896,1.065) | 0.998 (0.990,1.005) | 0.999 (0.995,1.003) | 0.998 (0.991,1.006) |
| Lower middle | 2,749 / 5,450 (50.44%) | 0.993 (0.923,1.068) | 0.986 (0.900,1.081) | 1.003 (0.899,1.118) | 1.002 (0.992,1.013) | 1.000 (0.995,1.005) | 0.999 (0.991,1.007) |
| Low | 1,483 / 2,226 (66.62%) | 1.084 (1.034,1.136)^**^ | 1.074 (1.009,1.143)^*^ | 1.094 (1.019,1.174)^*^ | 1.009 (1.002,1.016)^**^ | 1.005 (1.001,1.008)^**^ | 1.007 (1.002,1.012)^*^ |
| p-value for interaction**^†^** |  | 0.277 | 0.673 | 0.263 | 0.092 | 0.199 | 0.442 |
| **Lifestyle categories** |  |  |  |  |  |  |  |
| Most healthy | 3,807 / 7,558 (50.37 %) | 1.037 (1.005,1.070)^*^ | 1.034 (0.993,1.077) | 1.040 (0.991,1.091) | 1.005 (1.001,1.009)^*^ | 1.002 (1.000,1.004)^*^ | 1.003 (0.999,1.007) |
| Moderately healthy | 2,733 / 5,429 (50.34 %) | 1.020 (0.983,1.059) | 1.020 (0.973,1.069) | 1.018 (0.963,1.077) | 1.001 (0.995,1.006) | 1.001 (0.998,1.003) | 1.002 (0.997,1.006) |
| Least healthy | 2,238 / 4,400 (50.86 %) | 1.031 (0.992,1.071) | 1.054 (1.002,1.109)^*^ | 1.002 (0.943,1.064) | 1.002 (0.996,1.007) | 1.002 (0.999,1.004) | 1.004 (1.000,1.009) |
| p-value for interaction**^†^** |  | 0.929 | 0.497 | 0.398 | 0.399 | 0.916 | 0.595 |

Note:

Abbreviations: SES, socioeconomic status; PM_2.5_, fine particulate matter with diameter < 2.5 μm; NO_2_, nitrogen dioxide; NO_X_, nitrogen oxides; NO, nitric oxide, NO was determined as the difference between NO_X_ and NO_2_

^*^p-value < 0.05; ^**^ p-value < 0.01; ^***^ p-value < 0.001.

**^†^**p-value for interaction: Multiplicative interaction was evaluated using hazard ratios for the product term between the SES (convert to integer: 1-5), lifestyle categories (convert to integer: 1-3) and major pollutant concentrations (μg/m^3^).

Single-full model: adjusted for age (except for stratification by age), sex (except for stratification by sex), SES (except for stratification by SES), BMI categories and lifestyle categories (except for stratification by lifestyle categories).

**Table S5.** The results of fully adjusted association models for asthma incidence, stratified by each quartile of PM_2.5_ (United Kingdom, 2006-2022).

| **Variable** | **PM_2.5_ [0.00, 0.25] [960 / 44,681 (2.15%)]** | | **PM_2.5_ (0.25, 0.50] [1,074 / 44,325 (2.42%)]** | | **PM_2.5_ (0.50, 0.75] [1,131 / 43,890 (2.58%)]** | | **PM_2.5_ (0.75, 1.00] [1,149 / 43,904 (2.62%)]** | | **p-value for interaction^†^** |
| --- | --- | --- | --- | --- | --- | --- | --- | --- | --- |
|  | **No. of events / Total (%)** | **HR (95% CI)** | **No. of events / Total (%)** | **HR (95% CI)** | **No. of events / Total (%)** | **HR (95% CI)** | **No. of events / Total (%)** | **HR (95% CI)** |  |
| SES |  |  |  |  |  |  |  |  | 0.272 |
| High | 258 / 15,148 (1.70%) | Ref. | 266 / 13,651 (1.95%) | Ref. | 267 / 13,105 (2.04%) | Ref. | 280 / 14,088 (1.99%) | Ref. |  |
| Upper middle | 300 / 12,516 (2.40%) | 1.330 (1.125,1.571)^**^ | 339 / 13,763 (2.46%) | 1.191 (1.014,1.400)^*^ | 387 / 14,583 (2.65%) | 1.219 (1.042,1.425)^*^ | 359 / 13,992 (2.57%) | 1.171 (1.001,1.371)^*^ |  |
| Middle | 146 / 7,579 (1.93%) | 1.160 (0.947,1.421) | 126 / 6,285 (2.00%) | 1.037 (0.839,1.282) | 143 / 5,371 (2.66%) | 1.300 (1.061,1.592)^*^ | 134 / 5,427 (2.47%) | 1.250 (1.017,1.536)^*^ |  |
| Lower middle | 103 / 4,406 (2.34%) | 1.330 (1.058,1.673)^*^ | 143 / 4,622 (3.09%) | 1.515 (1.235,1.858)^***^ | 118 / 4,232 (2.79%) | 1.281 (1.031,1.592)^*^ | 98 / 3,401 (2.88%) | 1.325 (1.052,1.669)^*^ |  |
| Low | 153 / 5,032 (3.04%) | 1.671 (1.365,2.045)^***^ | 200 / 6,004 (3.33%) | 1.577 (1.310,1.898)^***^ | 216 / 6,599 (3.27%) | 1.435 (1.197,1.720)^***^ | 278 / 6,996 (3.97%) | 1.800 (1.522,2.129)^***^ |  |
| Lifestyle categories |  |  |  |  |  |  |  |  | 0.173 |
| Most healthy | 427 / 20,872 (2.05%) | Ref. | 504 / 20,122 (2.50%) | Ref. | 503 / 19,420 (2.59%) | Ref. | 433 / 18,412 (2.35%) | Ref. |  |
| Moderately healthy | 283 / 13,561 (2.09%) | 1.024 (0.879,1.192) | 318 / 13,671 (2.33%) | 0.932 (0.809,1.074) | 347 / 13,496 (2.57%) | 0.991 (0.863,1.138) | 368 / 13,746 (2.68%) | 1.125 (0.978,1.294) |  |
| Least healthy | 250 / 10,248 (2.44%) | 1.208 (1.027,1.422)^*^ | 252 / 10,532 (2.39%) | 0.964 (0.824,1.128) | 281 / 10,974 (2.56%) | 1.011 (0.868,1.177) | 348 / 11,746 (2.96%) | 1.223 (1.056,1.416)^**^ |  |
| Exposure to second-hand smoke |  |  |  |  |  |  |  |  | **0.014** |
| No | 767 / 36,857 (2.08%) | Ref. | 828 / 35,423 (2.34%) | Ref. | 851 / 34,136 (2.49%) | Ref. | 759 / 32,700 (2.32%) | Ref. |  |
| Yes(≥1 h/w) | 193 / 7,824 (2.47%) | 1.122 (0.956,1.316) | 246 / 8,902 (2.76%) | 1.137 (0.985,1.313) | 280 / 9,754 (2.87%) | 1.110 (0.969,1.273) | 390 / 11,204 (3.48%) | 1.397 (1.234,1.581)^***^ |  |
| Smoking status |  |  |  |  |  |  |  |  | 0.579 |
| Never | 576 / 28,481 (2.02%) | Ref. | 659 / 28,238 (2.33%) | Ref. | 688 / 27,494 (2.50%) | Ref. | 647 / 26,230 (2.47%) | Ref. |  |
| Previous/Current | 384 / 16,200 (2.37%) | 1.142 (1.002,1.301)^*^ | 415 / 16,087 (2.58%) | 1.131 (0.997,1.283) | 443 / 16,396 (2.70%) | 1.058 (0.938,1.193) | 502 / 17,674 (2.84%) | 1.164 (1.033,1.311)^*^ |  |

Note:

Abbreviations: PM_2.5_, fine particulate matter with diameter < 2.5 μm; Ref., reference; SES, socioeconomic status; BMI, body mass index (calculated as weight in kilograms divided by height in meters squared).

^*^p-value < 0.05; ^**^ p-value < 0.01; ^***^ p-value < 0.001.

**^†^**p-value for interaction: Multiplicative interaction was evaluated using hazard ratios for the product term between the SES (convert to integer: 1-5), lifestyle categories (convert to integer: 1-3) and PM_2.5_ concentration (μg/m^3^).

Fully adjusted association model: adjusted for age, sex, SES, BMI categories and lifestyle categories.

**Table S6.** The results of fully adjusted association models for asthma hospitalization, stratified by each quartile of PM_2.5_ (United Kingdom, 2006-2022).

| **Variable** | **PM_2.5_ [0.00, 0.25] [2,123 / 4,380 (48.47%)]** | | **PM_2.5_ (0.25, 0.50] [2,180 / 4,329 (50.36%)]** | | **PM_2.5_ (0.50, 0.75] [2,241 / 4,332 (51.73%)]** | | **PM_2.5_ (0.75, 1.00] [2,234 / 4,346 (51.40%)]** | | **p-value for interaction^†^** |
| --- | --- | --- | --- | --- | --- | --- | --- | --- | --- |
|  | **No. of events / Total (%)** | **HR (95% CI)** | **No. of events / Total (%)** | **HR (95% CI)** | **No. of events / Total (%)** | **HR (95% CI)** | **No. of events / Total (%)** | **HR (95% CI)** |  |
| SES |  |  |  |  |  |  |  |  | 0.277 |
| High | 676 / 1,713 (39.46%) | Ref. | 648 / 1,527 (42.44%) | Ref. | 621 / 1,445 (42.98%) | Ref. | 648 / 1,533 (42.27%) | Ref. |  |
| Upper middle | 608 / 1,239 (49.07%) | 1.322 (1.184,1.476)^***^ | 691 / 1,387 (49.82%) | 1.210 (1.087,1.348)^**^ | 734 / 1,441 (50.94%) | 1.204 (1.081,1.341)^**^ | 716 / 1,383 (51.77%) | 1.272 (1.142,1.416)^***^ |  |
| Middle | 348 / 632 (55.06%) | 1.429 (1.244,1.642)^***^ | 308 / 588 (52.38%) | 1.167 (1.011,1.346)^*^ | 272 / 503 (54.08%) | 1.239 (1.067,1.438)^**^ | 253 / 498 (50.80%) | 1.200 (1.033,1.393)^*^ |  |
| Lower middle | 200 / 344 (58.14%) | 1.533 (1.296,1.813)^***^ | 206 / 332 (62.05%) | 1.393 (1.178,1.647)^***^ | 209 / 336 (62.20%) | 1.480 (1.252,1.750)^***^ | 157 / 260 (60.38%) | 1.429 (1.190,1.716)^***^ |  |
| Low | 291 / 452 (64.38%) | 1.765 (1.519,2.050)^***^ | 327 / 495 (66.06%) | 1.615 (1.398,1.864)^***^ | 405 / 607 (66.72%) | 1.732 (1.510,1.988)^***^ | 460 / 672 (68.45%) | 1.842 (1.620,2.095)^***^ |  |
| Lifestyle categories |  |  |  |  |  |  |  |  | 0.929 |
| Most healthy | 965 / 2,011 (47.99%) | Ref. | 984 / 1,947 (50.54%) | Ref. | 997 / 1,893 (52.67%) | Ref. | 861 / 1,707 (50.44%) | Ref. |  |
| Moderately healthy | 675 / 1,354 (49.85%) | 1.101 (0.996,1.216) | 640 / 1,297 (49.34%) | 0.991 (0.896,1.096) | 691 / 1,376 (50.22%) | 0.967 (0.876,1.067) | 727 / 1,402 (51.85%) | 1.064 (0.964,1.175) |  |
| Least healthy | 483 / 1,015 (47.59%) | 1.026 (0.916,1.148) | 556 / 1,085 (51.24%) | 1.080 (0.969,1.204) | 553 / 1,063 (52.02%) | 1.030 (0.925,1.147) | 646 / 1,237 (52.22%) | 1.105 (0.994,1.227) |  |
| Exposure to second-hand smoke |  |  |  |  |  |  |  |  | 0.705 |
| No | 1,711 / 3,547 (48.24%) | Ref. | 1,636 / 3,322 (49.25%) | Ref. | 1,650 / 3,265 (50.54%) | Ref. | 1,551 / 3,096 (50.10%) | Ref. |  |
| Yes(≥1 h/w) | 412 / 833 (49.46%) | 1.017 (0.912,1.134) | 544 / 1,007 (54.02%) | 1.139 (1.033,1.257)^**^ | 591 / 1,067 (55.39%) | 1.087 (0.987,1.196) | 683 / 1,250 (54.64%) | 1.073 (0.979,1.175) |  |
| Smoking status |  |  |  |  |  |  |  |  | 0.289 |
| Never | 1,333 / 2,808 (47.47%) | Ref. | 1,374 / 2,775 (49.51%) | Ref. | 1,391 / 2,741 (50.75%) | Ref. | 1,334 / 2,596 (51.39%) | Ref. |  |
| Previous/Current | 790 / 1,572 (50.25%) | 1.036 (0.948,1.133) | 806 / 1,554 (51.87%) | 1.035 (0.947,1.130) | 850 / 1,591 (53.43%) | 1.070 (0.980,1.168) | 900 / 1,750 (51.43%) | 0.999 (0.916,1.089) |  |

Note:

Abbreviations: PM_2.5_, fine particulate matter with diameter < 2.5 μm; Ref., reference; SES, socioeconomic status; BMI, body mass index (calculated as weight in kilograms divided by height in meters squared).

^*^p-value < 0.05; ^**^ p-value < 0.01; ^***^ p-value < 0.001.

**^†^**p-value for interaction: Multiplicative interaction was evaluated using hazard ratios for the product term between the SES (convert to integer: 1-5), lifestyle categories (convert to integer: 1-3) and PM_2.5_ concentration (μg/m^3^).

Fully adjusted association model: adjusted for age, sex, SES, BMI categories and lifestyle categories.

**Table S7.** Sensitivity analysis: Associations of the socioeconomic status (individual, county level, or both) with two primary asthma-related outcomes in the fully adjusted association models (United Kingdom, 2006-2022).

| **Variable** | **Asthma-onset cohort (n = 176,800)** | | | **Asthma-hospitalisation cohort (n = 17,387)** | | |
| --- | --- | --- | --- | --- | --- | --- |
|  | **SES-adjusted HR (95% CI)^a^** | **Townsend deprivation index-adjusted HR (95%CI)^b^** | **Both-adjusted HR (95%CI)^c^** | **SES-adjusted HR (95% CI)^a^** | **Townsend deprivation index-adjusted HR (95%CI)^b^** | **Both-adjusted HR (95%CI)^c^** |
| SES |  |  |  |  |  |  |
| High | Ref. | -^†^ | Ref. | Ref. | -^†^ | Ref. |
| Upper middle | 1.225 (1.131,1.328)^***^ | -^†^ | 1.215 (1.121,1.316)^***^ | 1.251 (1.186,1.321)^***^ | -^†^ | 1.250 (1.184,1.320)^***^ |
| Middle | 1.182 (1.066,1.310)^**^ | -^†^ | 1.153 (1.035,1.284)^*^ | 1.263 (1.175,1.357)^***^ | -^†^ | 1.259 (1.171,1.353)^***^ |
| Lower middle | 1.377 (1.234,1.536)^***^ | -^†^ | 1.340 (1.194,1.505)^***^ | 1.460 (1.341,1.590)^***^ | -^†^ | 1.458 (1.339,1.588)^***^ |
| Low | 1.622 (1.481,1.777)^***^ | -^†^ | 1.549 (1.403,1.710)^***^ | 1.744 (1.627,1.870)^***^ | -^†^ | 1.726 (1.610,1.851)^***^ |
| Townsend deprivation index (per unit) | -^†^ | 1.028 (1.016,1.039)^***^ | 1.021 (1.009,1.033)^***^ | -^†^ | 1.016 (1.008,1.024)^***^ | 1.009 (1.001,1.017)^*^ |
| PM_2.5_ (1 μg/m^3^) | 1.064 (1.034,1.094)^***^ | 1.041 (1.009,1.074)^*^ | 1.040 (1.008,1.073)^*^ | 1.031 (1.010,1.052)^**^ | 1.024 (1.001,1.047)^*^ | 1.021 (0.998,1.044) |
| SES |  |  |  |  |  |  |
| High | Ref. | -^†^ | Ref. | Ref. | -^†^ | Ref. |
| Upper middle | 1.224 (1.130,1.326)^***^ | -^†^ | 1.213 (1.119,1.314)^***^ | 1.252 (1.186,1.321)^***^ | -^†^ | 1.248 (1.183,1.318)^***^ |
| Middle | 1.179 (1.063,1.307)^**^ | -^†^ | 1.151 (1.033,1.282)^*^ | 1.265 (1.177,1.359)^***^ | -^†^ | 1.261 (1.173,1.356)^***^ |
| Lower middle | 1.374 (1.231,1.533)^***^ | -^†^ | 1.339 (1.193,1.503)^***^ | 1.460 (1.341,1.590)^***^ | -^†^ | 1.457 (1.338,1.587)^***^ |
| Low | 1.623 (1.481,1.778)^***^ | -^†^ | 1.547 (1.401,1.707)^***^ | 1.747 (1.630,1.872)^***^ | -^†^ | 1.727 (1.610,1.852)^***^ |
| Townsend deprivation index (per unit) | -^†^ | 1.029 (1.018,1.040)^***^ | 1.022 (1.011,1.033)^***^ | -^†^ | 1.017 (1.009,1.025)^***^ | 1.010 (1.002,1.017)^*^ |
| Traffic PM_2.5_ (1 μg/m^3^) | 1.082 (1.043,1.123)^***^ | 1.060 (1.019,1.102)^**^ | 1.056 (1.015,1.098)^**^ | 1.035 (1.009,1.063)^*^ | 1.029 (1.001,1.059)^*^ | 1.024 (0.996,1.053) |
| SES |  |  |  |  |  |  |
| High | Ref. | -^†^ | Ref. | Ref. | -^†^ | Ref. |
| Upper middle | 1.230 (1.136,1.333)^***^ | -^†^ | 1.215 (1.121,1.317)^***^ | 1.254 (1.188,1.324)^***^ | -^†^ | 1.251 (1.185,1.320)^***^ |
| Middle | 1.174 (1.059,1.302)^**^ | -^†^ | 1.149 (1.032,1.280)^*^ | 1.263 (1.175,1.357)^***^ | -^†^ | 1.259 (1.171,1.353)^***^ |
| Lower middle | 1.373 (1.231,1.532)^***^ | -^†^ | 1.340 (1.194,1.504)^***^ | 1.463 (1.344,1.593)^***^ | -^†^ | 1.459 (1.340,1.589)^***^ |
| Low | 1.637 (1.494,1.793)^***^ | -^†^ | 1.551 (1.405,1.712)^***^ | 1.755 (1.638,1.881)^***^ | -^†^ | 1.728 (1.612,1.854)^***^ |
| Townsend deprivation index (per unit) | -^†^ | 1.034 (1.023,1.045)^***^ | 1.027 (1.016,1.038)^***^ | -^†^ | 1.019 (1.012,1.027)^***^ | 1.012 (1.004,1.019)^**^ |
| Non-Traffic PM_2.5_ (1 μg/m^3^) | 1.038 (0.993,1.085) | 1.006 (0.961,1.054) | 1.009 (0.964,1.056) | 1.023 (0.992,1.056) | 1.011 (0.979,1.044) | 1.012 (0.980,1.045) |
| SES |  |  |  |  |  |  |
| High | Ref. | -^†^ | Ref. | Ref. | -^†^ | Ref. |
| Upper middle | 1.230 (1.135,1.332)^***^ | -^†^ | 1.215 (1.121,1.316)^***^ | 1.254 (1.188,1.324)^***^ | -^†^ | 1.249 (1.184,1.319)^***^ |
| Middle | 1.176 (1.061,1.304)^**^ | -^†^ | 1.151 (1.033,1.282)^*^ | 1.264 (1.176,1.359)^***^ | -^†^ | 1.260 (1.172,1.355)^***^ |
| Lower middle | 1.372 (1.230,1.530)^***^ | -^†^ | 1.340 (1.193,1.504)^***^ | 1.463 (1.343,1.593)^***^ | -^†^ | 1.458 (1.339,1.588)^***^ |
| Low | 1.637 (1.495,1.793)^***^ | -^†^ | 1.551 (1.405,1.712)^***^ | 1.756 (1.639,1.882)^***^ | -^†^ | 1.729 (1.612,1.854)^***^ |
| Townsend deprivation index (per unit) | -^†^ | 1.034 (1.023,1.045)^***^ | 1.027 (1.016,1.038)^***^ | -^†^ | 1.020 (1.012,1.027)^***^ | 1.011 (1.004,1.019)^**^ |
| PM_10_ (1 μg/m^3^) | 1.009 (0.993,1.025) | 1.001 (0.985,1.018) | 1.001 (0.985,1.017) | 1.005 (0.994,1.016) | 1.002 (0.991,1.014) | 1.001 (0.990,1.013) |
| SES |  |  |  |  |  |  |
| High | Ref. | -^†^ | Ref. | Ref. | -^†^ | Ref. |
| Upper middle | 1.231 (1.136,1.333)^***^ | -^†^ | 1.215 (1.121,1.316)^***^ | 1.255 (1.189,1.324)^***^ | -^†^ | 1.249 (1.184,1.319)^***^ |
| Middle | 1.175 (1.060,1.303)^**^ | -^†^ | 1.150 (1.033,1.281)^*^ | 1.264 (1.176,1.359)^***^ | -^†^ | 1.260 (1.172,1.355)^***^ |
| Lower middle | 1.371 (1.229,1.530)^***^ | -^†^ | 1.339 (1.193,1.504)^***^ | 1.463 (1.344,1.594)^***^ | -^†^ | 1.458 (1.339,1.588)^***^ |
| Low | 1.639 (1.497,1.795)^***^ | -^†^ | 1.550 (1.404,1.711)^***^ | 1.759 (1.641,1.885)^***^ | -^†^ | 1.729 (1.613,1.855)^***^ |
| Townsend deprivation index (per unit) | -^†^ | 1.035 (1.024,1.045)^***^ | 1.027 (1.017,1.038)^***^ | -^†^ | 1.020 (1.013,1.027)^***^ | 1.012 (1.004,1.019)^**^ |
| PM_coarse_ (1 μg/m^3^) | 0.998 (0.965,1.031) | 0.988 (0.955,1.022) | 0.989 (0.956,1.023) | 1.000 (0.977,1.023) | 0.998 (0.975,1.022) | 0.997 (0.974,1.020) |
| SES |  |  |  |  |  |  |
| High | Ref. | -^†^ | Ref. | Ref. | -^†^ | Ref. |
| Upper middle | 1.232 (1.137,1.334)^***^ | -^†^ | 1.217 (1.123,1.319)^***^ | 1.255 (1.189,1.324)^***^ | -^†^ | 1.250 (1.184,1.319)^***^ |
| Middle | 1.182 (1.066,1.310)^**^ | -^†^ | 1.153 (1.035,1.284)^*^ | 1.264 (1.176,1.359)^***^ | -^†^ | 1.261 (1.173,1.355)^***^ |
| Lower middle | 1.383 (1.239,1.543)^***^ | -^†^ | 1.343 (1.196,1.508)^***^ | 1.465 (1.345,1.595)^***^ | -^†^ | 1.460 (1.340,1.589)^***^ |
| Low | 1.633 (1.490,1.788)^***^ | -^†^ | 1.554 (1.408,1.715)^***^ | 1.752 (1.635,1.878)^***^ | -^†^ | 1.730 (1.613,1.856)^***^ |
| Townsend deprivation index (per unit) | -^†^ | 1.031 (1.019,1.043)^***^ | 1.023 (1.011,1.035)^***^ | -^†^ | 1.020 (1.011,1.028)^***^ | 1.010 (1.002,1.019)^*^ |
| NO_2_ (1 μg/m^3^) | 1.007 (1.003,1.011)^***^ | 1.002 (0.998,1.007) | 1.003 (0.999,1.008) | 1.003 (1.000,1.006)^*^ | 1.000 (0.997,1.003) | 1.001 (0.998,1.004) |
| SES |  |  |  |  |  |  |
| High | Ref. | -^†^ | Ref. | Ref. | -^†^ | Ref. |
| Upper middle | 1.228 (1.134,1.331)^***^ | -^†^ | 1.216 (1.122,1.318)^***^ | 1.253 (1.188,1.323)^***^ | -^†^ | 1.250 (1.184,1.319)^***^ |
| Middle | 1.181 (1.065,1.309)^**^ | -^†^ | 1.153 (1.035,1.284)^*^ | 1.265 (1.176,1.359)^***^ | -^†^ | 1.261 (1.173,1.355)^***^ |
| Lower middle | 1.379 (1.236,1.539)^***^ | -^†^ | 1.342 (1.196,1.507)^***^ | 1.462 (1.343,1.593)^***^ | -^†^ | 1.459 (1.340,1.589)^***^ |
| Low | 1.629 (1.487,1.784)^***^ | -^†^ | 1.552 (1.406,1.713)^***^ | 1.749 (1.632,1.875)^***^ | -^†^ | 1.729 (1.613,1.855)^***^ |
| Townsend deprivation index (per unit) | -^†^ | 1.030 (1.018,1.041)^***^ | 1.022 (1.011,1.034)^***^ | -^†^ | 1.018 (1.010,1.026)^***^ | 1.010 (1.001,1.018)^*^ |
| NO_X_ (1 μg/m^3^) | 1.004 (1.002,1.006)^***^ | 1.002 (1.000,1.004) | 1.002 (1.000,1.004)^*^ | 1.002 (1.000,1.003)^*^ | 1.001 (0.999,1.002) | 1.001 (0.999,1.003) |
| SES |  |  |  |  |  |  |
| High | Ref. | -^†^ | Ref. | Ref. | -^†^ | Ref. |
| Upper middle | 1.226 (1.132,1.329)^***^ | -^†^ | 1.214 (1.120,1.315)^***^ | 1.252 (1.187,1.322)^***^ | -^†^ | 1.249 (1.183,1.318)^***^ |
| Middle | 1.179 (1.064,1.307)^**^ | -^†^ | 1.152 (1.034,1.283)^*^ | 1.265 (1.177,1.360)^***^ | -^†^ | 1.261 (1.173,1.356)^***^ |
| Lower middle | 1.374 (1.232,1.533)^***^ | -^†^ | 1.340 (1.193,1.504)^***^ | 1.460 (1.341,1.590)^***^ | -^†^ | 1.457 (1.338,1.587)^***^ |
| Low | 1.628 (1.486,1.783)^***^ | -^†^ | 1.549 (1.403,1.710)^***^ | 1.749 (1.632,1.874)^***^ | -^†^ | 1.728 (1.611,1.853)^***^ |
| Townsend deprivation index (per unit) | -^†^ | 1.030 (1.019,1.041)^***^ | 1.023 (1.012,1.034)^***^ | -^†^ | 1.018 (1.010,1.025)^***^ | 1.010 (1.002,1.017)^*^ |
| NO (1 μg/m^3^) | 1.006 (1.003,1.009)^***^ | 1.004 (1.001,1.007)^*^ | 1.004 (1.000,1.007)^*^ | 1.003 (1.001,1.005)^*^ | 1.002 (1.000,1.005) | 1.002 (0.999,1.005) |

Note:

Abbreviations: PM_2.5_, fine particulate matter with diameter < 2.5 μm; PM_10_, particulate matter with diameter < 10 μm; PM_coarse_, particulate matter with diameter between 2.5 μm and 10 μm; NO_2_, nitrogen dioxide; NO_X_, nitrogen oxides; NO, nitric oxide, NO was determined as the difference between NO_X_ and NO_2_; HR, hazard ratio; CI, confidence interval; Ref., reference; SES, socioeconomic status.

^*^p-value < 0.05; ^**^ p-value < 0.01; ^***^ p-value < 0.001.

^†^ The variable was not included in the model.

^a^Hazard ratio (HR) and 95% confidence interval (95% CI) of SES and ambient air pollutants for two primary asthma-related outcomes at follow-up.

^b^Hazard ratio (HR) and 95% confidence interval (95% CI) of Townsend deprivation index and ambient air pollutants for two primary asthma-related outcomes at follow-up.

^c^Hazard ratio (HR) and 95% confidence interval (95% CI) of SES, Townsend deprivation index and ambient air pollutants for two primary asthma-related outcomes at follow-up.

Fully adjusted association model: adjusted for age, sex, BMI categories and lifestyle categories.

**Table S8.** Sensitivity analysis: Associations between exposure to ambient air pollution and two primary asthma-related outcomes in the single-full model after limiting length of time at current address, follow-up time or time when the event occurred (United Kingdom, 2006-2022).

| **Cohort** | **Total** | | **Length of time at current address ≥ 3 years** | | **Follow-up time ≤ 3 years** | | **Excluded events that occurred ≤ 3 years** | |
| --- | --- | --- | --- | --- | --- | --- | --- | --- |
|  | **No. of events / Total (%)** | **HR (95%CI)** | **No. of events / Total (%)** | **HR (95%CI)** | **No. of events / Total (%)** | **HR (95%CI)** | **No. of events / Total (%)** | **HR (95%CI)** |
| **Asthma-onset cohort** | 4,314 / 176,800 (2.44%) |  | 3,957 / 162,258 (2.44%) |  | 693 / 176,800 (0.39%) |  | 3,621 / 176,107 (2.06%) |  |
| PM_2.5_ (1 μg/m^3^) |  | 1.064 (1.034,1.094)*** |  | 1.070 (1.039,1.103)*** |  | 1.063 (0.990,1.140) |  | 1.066 (1.034,1.099)*** |
| Traffic PM_2.5_ (1 μg/m^3^) |  | 1.082 (1.043,1.123)*** |  | 1.083 (1.042,1.126)*** |  | 1.097 (1.001,1.202)* |  | 1.080 (1.037,1.124)*** |
| Non-traffic PM_2.5_ (1 μg/m^3^) |  | 1.038 (0.993,1.085) |  | 1.047 (1.000,1.097) |  | 1.014 (0.908,1.133) |  | 1.043 (0.994,1.094) |
| PM_10_ (1 μg/m^3^) |  | 1.009 (0.993,1.025) |  | 1.008 (0.992,1.025) |  | 1.007 (0.969,1.048) |  | 1.009 (0.992,1.027) |
| PM_coarse_ (1 μg/m^3^) |  | 0.998 (0.965,1.031) |  | 0.996 (0.962,1.031) |  | 1.009 (0.929,1.095) |  | 0.995 (0.960,1.032) |
| NO_2_ (1 μg/m^3^) |  | 1.007 (1.003,1.011)*** |  | 1.008 (1.004,1.012)*** |  | 1.005 (0.995,1.015) |  | 1.008 (1.003,1.012)*** |
| NO_X_ (1 μg/m^3^) |  | 1.004 (1.002,1.006)*** |  | 1.004 (1.002,1.006)*** |  | 1.003 (0.999,1.008) |  | 1.004 (1.002,1.006)*** |
| NO (1 μg/m^3^) |  | 1.006 (1.003,1.009)*** |  | 1.006 (1.003,1.010)*** |  | 1.006 (0.999,1.014) |  | 1.006 (1.002,1.009)** |
| **Asthma-hospitalisation cohort** | 8,778 / 17,387 (50.49%) |  | 8,011 / 15,817 (50.65%) |  | 2,704 / 17,387 (15.55%) |  | 6,074 / 14,683 (41.37%) |  |
| PM_2.5_ |  | 1.031 (1.010,1.052)** |  | 1.031 (1.010,1.053)** |  | 1.038 (1.001,1.077)* |  | 1.027 (1.003,1.052)* |
| Traffic PM_2.5_ |  | 1.035 (1.009,1.063)* |  | 1.040 (1.012,1.069)** |  | 1.040 (0.991,1.090) |  | 1.033 (1.001,1.066)* |
| Non-traffic PM_2.5_ |  | 1.023 (0.992,1.056) |  | 1.019 (0.986,1.052) |  | 1.036 (0.979,1.095) |  | 1.018 (0.981,1.057) |
| PM_10_ |  | 1.005 (0.994,1.016) |  | 1.004 (0.993,1.016) |  | 1.013 (0.994,1.034) |  | 1.001 (0.988,1.014) |
| PM_coarse_ |  | 1.000 (0.977,1.023) |  | 1.001 (0.977,1.026) |  | 1.009 (0.968,1.052) |  | 0.996 (0.969,1.024) |
| NO_2_ |  | 1.003 (1.000,1.006)* |  | 1.003 (1.000,1.006) |  | 1.003 (0.998,1.008) |  | 1.003 (0.999,1.006) |
| NO_X_ |  | 1.002 (1.000,1.003)* |  | 1.002 (1.000,1.003)* |  | 1.002 (0.999,1.004) |  | 1.002 (1.000,1.003)* |
| NO |  | 1.003 (1.001,1.005)* |  | 1.003 (1.001,1.006)* |  | 1.003 (0.999,1.007) |  | 1.003 (1.000,1.006)* |

Note:

Abbreviations: PM_2.5_, fine particulate matter with diameter < 2.5 μm; PM_10_, particulate matter with diameter < 10 μm; PM_coarse_, particulate matter with diameter between 2.5 μm and 10 μm; NO_2_, nitrogen dioxide; NO_X_, nitrogen oxides; NO, nitric oxide, NO was determined as the difference between NO_X_ and NO_2_; HR, hazard ratio; CI, confidence interval; Ref., reference.

^*^p-value < 0.05; ^**^ p-value < 0.01; ^***^ p-value < 0.001.

Single-full model: adjusted for age, sex, SES, and BMI categories. SES, socioeconomic status; BMI, body mass index (calculated as weight in kilograms divided by height in meters squared).

**SUPPLEMENTAL FIGURES**

**Figure S1.** Two methods to regress PM_2.5_ on NO to estimate the amount of PM_2.5_ attributable to NO, identified as traffic-related PM_2.5_. (A) Linear regression; (B) Smooth Spline regression model (United Kingdom, 2006-2022).

Abbreviations: PM_2.5_, fine particulate matter with diameter < 2.5 μm; NO_2_, nitrogen dioxide; NO_X_, nitrogen oxides; NO, nitric oxide, NO was determined as the difference between NO_X_ and NO_2_.

**Figure S2.** Akaike information criterion (AIC) and Bayesian information criterion (BIC) of models with 2 to 7 latent classes (United Kingdom, 2006-2022).

**Figure S3.** Pairwise Spearman’s correlation between air pollutants across the two cohorts. **(A)** Five pollutant variables directly provided by UK Biobank; **(B)** PM_2.5_ and other calculated pollutant variables (United Kingdom, 2006-2022).

Note:

Abbreviations: PM_2.5_, fine particulate matter with diameter < 2.5 μm; PM_10_, particulate matter with diameter < 10 μm; PM_coarse_, particulate matter with diameter between 2.5 μm and 10 μm; NO_2_, nitrogen dioxide; NO_X_, nitrogen oxides; NO, nitric oxide, NO was determined as the difference between NO_X_ and NO_2_.

**Figure S4.** Kaplan-Meier survival curve for four asthma-related outcomes. **(A)** Asthma-onset; **(B)** Asthma-related hospitalizations (United Kingdom, 2006-2022).

**Figure S5.** Association between two primary asthma-related outcomes and all variables in the single-full model of non-traffic PM_2.5_ (United Kingdom, 2006-2022).

Note:

Abbreviations: PM_2.5_, fine particulate matter with diameter < 2.5 μm; HR, hazard ratio; CI, confidence interval; Ref., reference; SES, socioeconomic status; BMI, body mass index (calculated as weight in kilograms divided by height in meters squared).

^a^Hazard ratio (HR) and 95% confidence interval (95% CI) of factors for asthma incidence at follow-up.

^b^Hazard ratio (HR) and 95% confidence interval (95% CI) of factors for asthma hospitalization at follow-up.

^*^p-value < 0.05; ^**^ p-value < 0.01; ^***^ p-value < 0.001.

^†^ The variable didn’t survive in the final stepwise regression model, which was based on the AIC (Akaike Information Criterion) criterion.

Single-full model: adjusted for age, sex, SES, BMI categories and lifestyle categories.

**Figure S6.** The dose-response relationships between the concentrations of PM_2.5_, traffic PM_2.5_, non-traffic PM_2.5_ and the risk of **(A-C)** asthma onset and **(D-F)** hospitalization within the single-full model (United Kingdom, 2006-2022).

Note:

Abbreviations: PM_2.5_, fine particulate matter with diameter < 2.5 μm.

Single-full model: adjusted for age, sex, SES, and BMI categories. SES, socioeconomic status; BMI, body mass index (calculated as weight in kilograms divided by height in meters squared).

**Figure S7.** Hazard ratios (HRs) and 95% confidence intervals (95% CIs) of ambient air pollutants for two primary asthma-related outcomes in the two-pollutant models. Compared with the single-full model, two-pollutant models for PM_2.5_, traffic PM_2.5_, non-traffic PM_2.5_, PM_10,_ and PM_coarse_ further adjusted for NO_2_, NO_X_, NO respectively, while two-pollutant models for NO_2_, NO_X_, NO further adjusted for PM_2.5_, traffic PM_2.5_, non-traffic PM_2.5_, PM_10,_ PM_coarse_ respectively (United Kingdom, 2006-2022).

Note:

Abbreviations: PM_2.5_, fine particulate matter with diameter < 2.5 μm; PM_10_, particulate matter with diameter < 10 μm; PM_coarse_, particulate matter with diameter between 2.5 μm and 10 μm; NO_2_, nitrogen dioxide; NO_X_, nitrogen oxides; NO, nitric oxide, NO was determined as the difference between NO_X_ and NO_2_.

^*^p-value < 0.05; ^**^ p-value < 0.01; ^***^ p-value < 0.001.

Single-full model: adjusted for age, sex, SES, BMI categories and lifestyle categories.

**Figure S8.** Cox proportional hazard regression models for two outcomes of asthma hospitalization and lifestyle variables. **(A)** Association between two outcomes of asthma hospitalization and lifestyle variables without or with adjustment for other variables; **(B)** Association between two outcomes of asthma hospitalization and all factors in the single-full model of lifestyle categories (United Kingdom, 2006-2022).

Note:

Abbreviations: HR, hazard ratio; CI, confidence interval; Ref., reference; SES, socioeconomic status; BMI, body mass index (calculated as weight in kilograms divided by height in meters squared).

^a^Hazard ratio (HR) and 95% confidence interval (95% CI) of factors for first admission for asthma at follow-up.

^b^Hazard ratio (HR) and 95% confidence interval (95% CI) of factors for re-admission for asthma at follow-up.

^*^p-value < 0.05; ^**^ p-value < 0.01; ^***^ p-value < 0.001.

Single-1 model: unadjusted for other variables.

Single-full model: adjusted for age, sex, SES, and BMI categories.

**Figure S9.** Cox proportional hazard regression models for first admission for asthma and re-admission for asthma at follow-up. **(A)** Association between two outcomes of asthma hospitalization and ambient air pollutants under various model adjustments; **(B)** Association between two outcomes of asthma hospitalization and all variables in the single-full model of PM_2.5_ or traffic PM_2.5_ (United Kingdom, 2006-2022).

Note:

Abbreviations: PM_2.5_, fine particulate matter with diameter < 2.5 μm; PM_10_, particulate matter with diameter < 10 μm; PM_coarse_, particulate matter with diameter between 2.5 μm and 10 μm; NO_2_, nitrogen dioxide; NO_X_, nitrogen oxides; NO, nitric oxide, NO was determined as the difference between NO_X_ and NO_2_; HR, hazard ratio; CI, confidence interval; Ref., reference; SES, socioeconomic status; BMI, body mass index (calculated as weight in kilograms divided by height in meters squared).

^a^Hazard ratio (HR) and 95% confidence interval (95% CI) of factors for first admission for asthma at follow-up.

^b^Hazard ratio (HR) and 95% confidence interval (95% CI) of factors for re-admission for asthma at follow-up.

^*^p-value < 0.05; ^**^ p-value < 0.01; ^***^ p-value < 0.001.

^†^ The variable didn’t survive in the final stepwise regression model, which was based on the AIC (Akaike Information Criterion) criterion.

Single-1 model: unadjusted for other variables.

Single-2 model: adjusted for age, sex, SES, and BMI categories.

Single-full model: adjusted for age, sex, SES, BMI categories and lifestyle categories.

**Figure S10.** Association between two outcomes of asthma hospitalization and all variables in the single-full model of non-traffic PM_2.5_ (United Kingdom, 2006-2022).

Note:

Abbreviations: PM_2.5_, fine particulate matter with diameter < 2.5 μm; HR, hazard ratio; CI, confidence interval; Ref., reference; SES, socioeconomic status; BMI, body mass index (calculated as weight in kilograms divided by height in meters squared).

^a^Hazard ratio (HR) and 95% confidence interval (95% CI) of factors for first admission for asthma at follow-up.

^b^Hazard ratio (HR) and 95% confidence interval (95% CI) of factors for re-admission for asthma at follow-up.

^*^p-value < 0.05; ^**^ p-value < 0.01; ^***^ p-value < 0.001.

^†^ The variable didn’t survive in the final stepwise regression model, which was based on the AIC (Akaike Information Criterion) criterion.

Single-full model: adjusted for age, sex, SES, BMI categories and lifestyle categories.

**Figure S11.** The joint association of four factors (socioeconomic status, lifestyle categories, exposure to second-hand smoke, smoking status) and PM_2.5_ level with **(A)** asthma onset and **(B)** hospitalization in the single-full model (United Kingdom, 2006-2022).

Note:

Abbreviations: PM_2.5_, fine particulate matter with diameter < 2.5 μm; Ref., reference; SES, socioeconomic status.

ns: not significant; ^*^p-value < 0.05; ^**^ p-value < 0.01; ^***^ p-value < 0.001.

Single-full model: adjusted for age, sex, SES, BMI categories and lifestyle categories.
